# NAA-modified DNA oligonucleotides with zwitterionic backbones: stereoselective synthesis of A–T phosphoramidite building blocks

**DOI:** 10.3762/bjoc.11.8

**Published:** 2015-01-13

**Authors:** Boris Schmidtgall, Claudia Höbartner, Christian Ducho

**Affiliations:** 1 Department of Chemistry, University of Paderborn, Warburger Str. 100, 33 098 Paderborn, Germany; 2 Department of Pharmacy, Pharmaceutical and Medicinal Chemistry, Saarland University, Campus C2 3, 66 123 Saarbrücken, Germany; 3 Max-Planck-Institute for Biophysical Chemistry, Am Fassberg 11, 37 077 Göttingen, Germany; 4 Department of Chemistry, Institute of Organic and Biomolecular Chemistry, Georg-August-University Göttingen, Tammannstr. 2, 37 077 Göttingen, Germany

**Keywords:** backbone modifications, DNA, nucleic acids, oligonucleotides, stereoselective synthesis, zwitterions

## Abstract

Modifications of the nucleic acid backbone are essential for the development of oligonucleotide-derived bioactive agents. The NAA-modification represents a novel artificial internucleotide linkage which enables the site-specific introduction of positive charges into the otherwise polyanionic backbone of DNA oligonucleotides. Following initial studies with the introduction of the NAA-linkage at T–T sites, it is now envisioned to prepare NAA-modified oligonucleotides bearing the modification at X–T motifs (X = A, C, G). We have therefore developed the efficient and stereoselective synthesis of NAA-linked 'dimeric' A–T phosphoramidite building blocks for automated DNA synthesis. Both the (*S*)- and the (*R*)-configured NAA-motifs were constructed with high diastereoselectivities to furnish two different phosphoramidite reagents, which were employed for the solid phase-supported automated synthesis of two NAA-modified DNA oligonucleotides. This represents a significant step to further establish the NAA-linkage as a useful addition to the existing 'toolbox' of backbone modifications for the design of bioactive oligonucleotide analogues.

## Introduction

Oligonucleotides are important agents for a number of biomedical applications [1]. Thus, they are employed to exert antigene [2] or antisense [3] mechanisms as well as to trigger or inhibit RNA interference [4]. The capability of sequence-specific molecular recognition is a striking feature of nucleic acids, but their high polarity represents a significant hurdle for cellular uptake and leads to problematic pharmcokinetics. Furthermore, they are prone to nuclease-mediated degradation. As a consequence, it is of utmost importance to modify oligonucleotide structures using chemical or enzymatic methods in order to develop oligonucleotide-based drug candidates or biomedical chemical probes [5–6].

Native nucleic acids are connected by phosphate diesters as linking units, thus leading to a polyanionic backbone structure. Implications of this characteristic feature of nucleic acid architecture have been discussed by Westheimer [7] and Benner [8–9], among others. However, the accumulation of negative charges in the nucleic acid backbone is mainly responsible for their limited membrane penetration. Consequently, a significant number of artificial internucleotide linkages has been studied with the aim to manipulate the charge pattern in the backbone and to enhance nuclease stability. The electroneutral nucleic acid mimic 'peptide nucleic acid' (PNA) [10–12] is capable of sequence-specific binding to nucleic acids, but it was found to display limited water solubility and a peptide-like folding behaviour [8]. For the selective replacement of some phosphate linkages in otherwise native oligonucleotide structures, e.g., amide [13–20], triazole [21–22], phosphoramidate [23] and phosphate triester [24] moieties were reported alongside a considerable number of other modifications.

In comparison, the introduction of positive charges into the nucleic acid scaffold has found less attention. Positively charged units were attached to the 2'-hydroxy group or the nucleobase as a compensation for the presence of negative charges in the phosphate backbone [25–29]. In contrast to such zwitterionic, but densely charged systems, only very few attempts were made to replace the phosphate moiety by a positively charged motif [30], mainly by Bruice et al. [31–34]. Selective replacement of some phosphates with cationic motifs furnished oligonucleotide 'chimera' [30] with zwitterionic backbone structures as reported both by Bruice [35–36] and Letsinger [37].

**Figure 1:**
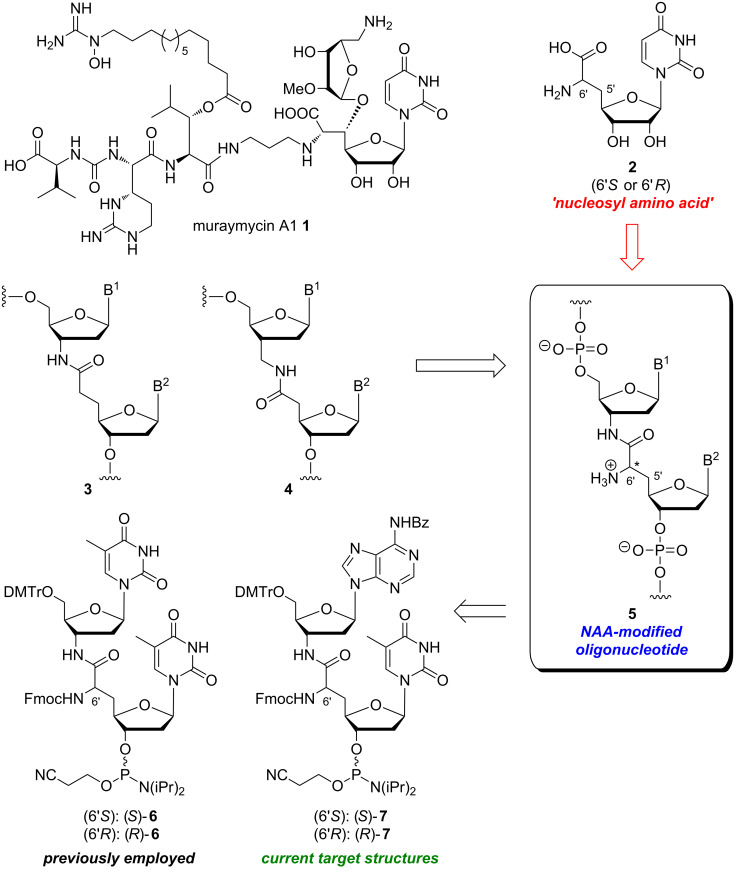
Design concept of nucleosyl amino acid (NAA)-modified oligonucleotides **5** formally derived from structures **1**–**4** (B^1^, B^2^ = nucleobases); previously employed 'dimeric' T–T phosphoramidites **6** [38] for the automated synthesis of NAA-modified oligonucleotides; new 'dimeric' A–T phosphoramidites **7** as target structures of this study (DMTr = 4,4'-dimethoxytrityl).

We have found the approach to prepare oligonucleotides with zwitterionic backbones interesting both for its fundamental implications and also for its potential contribution to the existing 'toolbox' of backbone modifications for bioactive oligonucleotide analogues. These considerations have led to our recently reported design of a novel artificial internucleotide linkage named 'NAA-modification' (Figure 1) [38]. Ongoing synthetic and structure-activity relationship (SAR) studies on naturally occurring muraymycin antibiotics (e.g., muraymycin A1 (**1**)) [39–46] have led to our previously reported synthesis of 'nucleosyl amino acid' structures **2** [47–48] as simplified 5'-defunctionalized analogues of the muraymycin core motif. Formally merging the **n**ucleosyl **a**mino **a**cid (NAA) structure of type **2** with previously reported amide internucleotide linkages such as **3** and **4** provides an 'NAA-modified oligonucleotide' **5**, i.e., a nucleic acid strand with the NAA-linkage replacing a phosphate diester motif. The amino group of the NAA-modification is expected to display a positive charge at physiological pH values, thus leading to a (partially) zwitterionic backbone structure in NAA-modified oligonucleotides.

We have previously described that NAA-modified DNA oligonucleotides can be obtained by standard solid phase-supported automated DNA synthesis, using the 'dimeric' phosphoramidite building blocks **6** (Figure 1) [38]. Overall, 24 different oligonucleotide sequences with one to four NAA-modifications at various positions were synthesized. The stereochemistry of the NAA-motif was either (*S*) or (*R*) (obtained by application of the corresponding phosphoramidites (*S*)-**6** or (*R*)-**6** for DNA synthesis) in order to study the influence of the spatial orientation of the positive charge. Melting temperature measurements showed that NAA-modified DNA oligonucleotides formed stable duplexes with native unmodified DNA or RNA counterstrands, although moderate destabilization in comparison to native duplexes was observed (particularly for DNA/RNA duplexes). Further experiments with native counterstrands bearing one nucleobase mismatch were performed, and duplex structures were studied by CD spectroscopy. Overall, we found that NAA-modified DNA oligonucleotides (i) formed stable duplexes with complementary counterstrands; (ii) were fully capable of mismatch discrimination and (iii) formed duplexes without significant structural distortion, i.e., B-form helices (DNA/DNA duplexes) and A-form helices (DNA/RNA duplexes), respectively. It was concluded that typical chemical properties of nucleic acids are retained in NAA-modified DNA oligonucleotides [38], thus making the NAA-linkage an interesting structural motif for oligonucleotide analogues.

Using 'dimeric' T–T phosphoramidites **6**, it was only possible to introduce the NAA-modification at T–T motifs. While this was fully sufficient for initial studies, it will be of major importance to develop methods for the synthesis of NAA-modified oligonucleotides with the NAA-motif at more variable positions in the base sequence. The synthesis of 'dimeric' X–T phosphoramidites (X = A, C, G) would enable an introduction of the NAA-linkage at every position in an oligonucleotide sequence with a T in 3'-direction, thus significantly broadening the applicability of the modification. In this work, we describe the stereoselective synthesis of 'dimeric' NAA-linked A–T phosphoramidites (*S*)-**7** and (*R*)-**7** (Figure 1) as well as their application in automated DNA synthesis. This represents the first step towards a comprehensive set of 'dimeric' NAA-linked X–T phosphoramidites for the automated chemical synthesis of NAA-modified DNA oligonucleotides.

## Results and Discussion

**Scheme 1:**
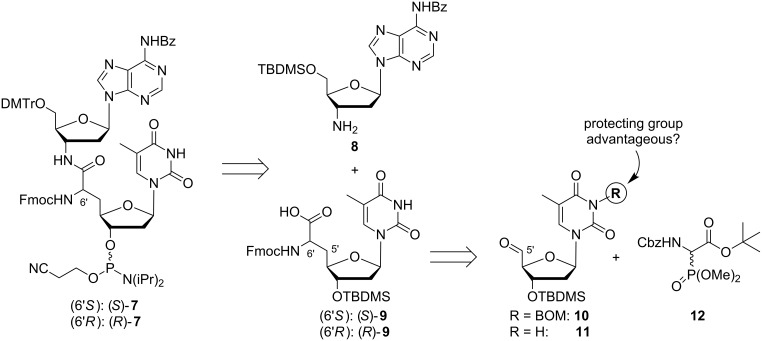
Retrosynthetic analysis of target phosphoramidites (*S*)-**7** and (*R*)-**7** (BOM = benzyloxymethyl).

For the synthesis of target phosphoramidites **7**, it was planned to employ a similar synthetic strategy as previously described by us for T–T phosphoramidites **6** [38]. One important objective was to construct the 6'-stereocenter of the NAA-linkage in a controlled fashion and to retain the resultant (6'*S*)- or the (6'*R*)-configuration, respectively, on the way to dimeric structures of type **7** (Scheme 1). Thus, it was envisioned that target compounds (*S*)-**7** and (*R*)-**7** could be obtained from protected 3'-amino-2',3'-dideoxyadenosine **8** and the *N*-Fmoc-protected thymidine-derived nucleosyl amino acids (*S*)-**9** and (*R*)-**9**, respectively, via amide coupling, protecting group manipulation and phosphitylation. The stereoselective synthesis of both 6'-epimers of nucleosyl amino acid **9** has been reported before. We have used 3-(*N*-BOM)-protected thymidine-5'-aldehyde **10** (BOM = benzyloxymethyl), which can readily be obtained from thymidine, in a sequence of Wittig–Horner reaction, asymmetric hydrogenation of the resultant didehydro nucleosyl amino acid and protecting group manipulations in order to obtain both 6'-epimers of **9** dependent on the choice of the hydrogenation catalyst [38]. Here, we report on further studies directed towards a possible synthesis of **9** without 3-(*N*-BOM)-protection of the thymine nucleobase. In the case of the corresponding uridine-derived nucleosyl amino acids, we have found that uracil protection was not advantageous for the aforementioned reaction sequence [48]. We have therefore decided to employ both the 3-(*N*-BOM)-protected thymidine-5'-aldehyde **10** and also its thymine-unprotected congener **11** in Wittig–Horner reactions with glycine-derived phosphonate **12** and to compare both possible routes towards **9**, i.e., with or without thymine protection (Scheme 1).

**Scheme 2:**
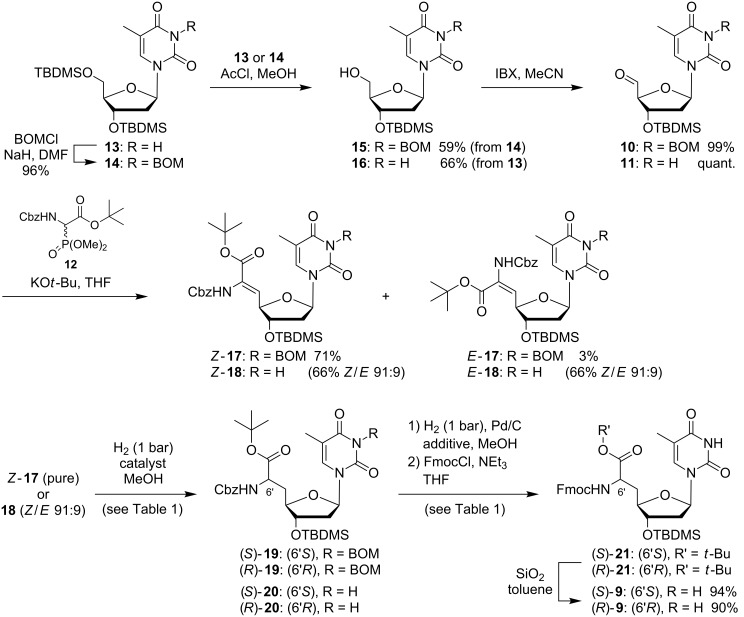
Synthesis of *N*-Fmoc-protected thymidine-derived nucleosyl amino acids (*S*)-**9** and (*R*)-**9**; details on the reactions from **17** and **18** to **21** (asymmetric hydrogenation and subsequent protecting group manipulations) are given in Table 1.

For the synthesis of the *N*-Fmoc-protected thymidine-derived nucleosyl amino acids (*S*)-**9** and (*R*)-**9**, 3',5'-bis-*O*-silylated thymidine **13** (which can be readily prepared from thymidine with TBDMS chloride and imidazole in pyridine as solvent in quantitative yield) was 3-*N*-protected by alkylation with benzyloxymethyl chloride (BOMCl), furnishing product **14** in 96% yield (Scheme 2). Although the route involving thymdine protection has been published before [38], it is also depicted in Scheme 2 in the interest of clarity and readability. Both bis-silyl ethers **13** and **14** then underwent selective acidic cleavage in the 5'-position using the conditions reported by Khan and Mondal [49], i.e., hydrochloric acid in methanol (generated by the reaction of acetyl chloride with methanol), thus providing 3'-*O*-TBDMS-protected derivatives **15** and **16** in yields of 59% and 66%, respectively. This method turned out to be advantageous compared to 5'-*O*-desilylation mediated by TFA, which had provided satisfying results in the case of the corresponding uridine derivatives [48]. The yield of the desired 5'-alcohols **15** and **16** was limited though by partial concomitant cleavage of the 3'-*O*-TBDMS group upon prolonged reaction times. Alcohols **15** and **16** were then oxidized to aldehydes **10** and **11** in quantitative yields using IBX in refluxing acetonitrile [50]. With respect to their limited stability, thymidine-5'-aldehydes **10** and **11** were not stored, but directly used for the subsequent Wittig–Horner reaction. They were therefore converted with glycine-derived phosphonate **12** [51–54] in the presence of potassium *tert*-butoxide as a base. As anticipated [47–48,55], these reactions showed pronounced stereoselectivity towards the *Z*-configured didehydro nucleosyl amino acids. In the case of the reaction of 3-(*N*-BOM)-protected thymidine-5'-aldehyde **10** with phosphonate **12**, isomer *Z*-**17** was isolated in 71% yield, with *E*-**17** representing a minor byproduct (3% yield) which could be separated by column chromatography. The assignment of the configuration of the newly formed trisubstituted C–C double bond was based on empirical ^1^H NMR criteria for didehydro amino acids [56], which were proven to be useful and reliable [38,47–48,57]. In the case of the conversion of 3-*N*-unprotected thymidine-5'-aldehyde **11** with phosphonate **12**, an unseparable mixture of *Z*-**18** and *E*-**18** was obtained in 66% overall yield (*Z*/*E* = 91:9). However, it is firmly established that the subsequent asymmetric hydrogenation proceeds significantly faster with the *Z*-configured didehydro amino acid substrate [58], and it was therefore decided to use the aforementioned mixture of double bond isomers (containing 9% of the unwanted *E*-isomer) as starting material for this transformation.

**Table 1:** Reactions from **17** and **18** to **21** (see Scheme 2).

		Asymmetric hydrogenation			Protecting group steps	
	
#	Starting material	Catalyst^a^	Reaction time	Yield^b^	Additive	Yield^c^

1	*Z*-**17**^d^	(*S*,*S*)-Me-DuPHOS-Rh	2 d	94% (*S*)-**19**	*n*-BuNH_2_	90% (*S*)-**21**
2	*Z*-**17**^d^	(*R*,*R*)-Me-DuPHOS-Rh	7 d	99% (*R*)-**19**	*n*-BuNH_2_	87% (*R*)-**21**
3	**18** (mix.)^e^	(*S*,*S*)-Me-DuPHOS-Rh	9 d	93% (*S*)-**20**	–	84% (*S*)-**21**
4	**18** (mix.)^e^	(*R*,*R*)-Me-DuPHOS-Rh	21 d	77% (*R*)-**20**	–	78% (*R*)-**21**

^a^Homogeneous chiral hydrogenation catalysts:
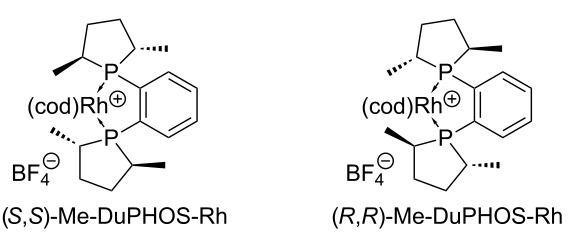
^b^d.r. >98:2 for all products; ^c^over 2 steps from **19** or **20**; ^d^pure *Z*-isomer; ^e^unseparable mixture of *Z*- and *E*-isomers (*Z*/*E* 91:9).

Asymmetric hydrogenation reactions were performed under homogeneous conditions using the chiral catalysts (*S*,*S*)-Me-DuPHOS-Rh or (*R*,*R*)-Me-DuPHOS-Rh, respectively [59]. It is known that asymmetric hydrogenations of *Z*-configured didehydro amino acids catalyzed by (*S*,*S*)-Me-DuPHOS-Rh give L-amino acids and that analogous reactions catalyzed by (*R*,*R*)-Me-DuPHOS-Rh provide D-amino acids [57,60]. This has also been observed when these catalysts were applied for the synthesis of uridine-derived nucleosyl amino acids [47–48]. It was therefore possible to direct the stereochemical outcome of the hydrogenation reaction by the choice of either the (*S*,*S*)- or the (*R*,*R*)-catalyst. As reported previously [38], the hydrogenation of pure 3-(*N*-BOM)-protected *Z*-**17** in the presence of (*S*,*S*)-Me-DuPHOS-Rh thus furnished thymidine-derived nucleosyl amino acid (*S*)-**19** in 94% yield, and its 6'-epimer (*R*)-**19** was obtained from the same starting material in 99% yield using catalytic amounts of (*R*,*R*)-Me-DuPHOS-Rh (Table 1, entries 1 and 2). Both transformations displayed excellent diastereoselectivies (d.r. >98:2 for both products), which indicated that these reactions proceeded in a catalyst-controlled fashion. The observation that full conversion of *Z*-**17** to (*S*)-**19** was reached after 2 days with the (*S*,*S*)-Me-DuPHOS-Rh catalyst, while completion of the reaction took 7 days with the (*R*,*R*)-Me-DuPHOS-Rh catalyst, suggested that the former transformation represented the apparent 'matched' case and the latter the apparent 'mismatched' case. This was also in agreement with similar findings for the synthesis of the according uridine-derived congeners [47–48]. When the isomeric mixture (*Z*/*E* = 91:9) of the 3-*N*-unprotected didehydro amino acid **18** was employed as starting material, 3-*N*-unprotected products (*S*)-**20** (93% yield, with (*S*,*S*)-Me-DuPHOS-Rh) and (*R*)-**20** (77% yield, with (*R*,*R*)-Me-DuPHOS-Rh) were obtained, again with excellent diastereoselectivities (d.r. >98:2 for both products, Table 1, entries 3 and 4). However, it was much more difficult to drive these reactions to completion as reflected by the significantly prolonged reaction times (9 and 21 days, respectively). For the apparent 'mismatched' case, i.e., hydrogenation of **18** in the presence of (*R*,*R*)-Me-DuPHOS-Rh, it was also necessary to increase the catalyst load (4 mol % added portionwise in comparison to 1–2 mol % for the other reactions), and even under these modified conditions, full conversion could not be reached, resulting in a moderately reduced yield. For both transformations of the isomeric mixture of **18**, no hydrogenation of the much less reactive *E*-isomer was observed, which is proven by the high diastereoselectivities. If one takes into account that the purity of the reactive *Z*-configured starting material *Z*-**18** was only 91% (vide supra), it is possible to calculate yields of ca. 100% and 85% for products (*S*)-**20** and (*R*)-**20**, respectively. Overall, it can still be concluded though that 3-*N*-unprotected didehydro amino acid **18** represented a less reactive substrate for the asymmetric hydrogenation key step, which was found to be problematic particularly for reactions on a larger scale (>1 g starting material). It is interesting to note that such limitations were not encountered when the analogous nucleobase-unprotected uridine-derived didehydro nucleosyl amino acid underwent asymmetric hydrogenation with the two aforementioned catalysts [48]. This decreased reactivity might have been the result of the presence of the *E*-isomer *E*-**18** in the reaction mixture, which probably led to partial inhibition of the Rh(I) catalyst.

In order to convert the hydrogenation products **19** and **20** into the desired building blocks **9**, three further transformations were necessary: (i) hydrogenolytic cleavage of the Cbz and, in the case of **19**, also of the BOM group; (ii) Fmoc-protection of the 6'-amino functionality (furnishing intermediates (*S*)-**21** and (*R*)-**21**) and (iii) cleavage of the *tert*-butyl ester (Scheme 2). Using the two diastereomerically pure 6'-epimers of 3-(*N*-BOM)-protected **19** as starting material, one challenge was to avoid unwanted side reactions resulting from the generation of formaldehyde in the reaction mixture. Hydrogenolysis of the BOM group affords toluene and formaldehyde as byproducts, and the Cbz-deprotected 6'-amino group can undergo unwanted reductive amination, i.e., methylation, with the liberated formaldehyde. Our method to prevent this side reaction was to include an excess of *n*-butylamine as an additive in the reaction mixture of the hydrogenolysis step. This way, the formaldehyde methylated the added *n*-butylamine, furnishing a reasonably volatile byproduct [38,48]. Subsequent Fmoc-protection under standard conditions then afforded diastereomerically pure products (*S*)-**21** (90% yield over 2 steps from (*S*)-**19**) and (*R*)-**21** (87% yield over 2 steps from (*R*)-**19**), respectively (Table 1, entires 1 and 2). For the analogous transformation of 3-*N*-unprotected (*S*)-**20** and (*R*)-**20**, it was possible to omit the additive *n*-butylamine, but yields were not improved due to this simplification (84% yield of (*S*)-**21** over 2 steps from (*S*)-**20**, 78% yield of (*R*)-**21** over 2 steps from (*R*)-**20**, Table 1, entries 3 and 4). Finally, the *tert*-butyl ester was cleaved selectively in the presence of the acid-labile silyl group using silica in refluxing toluene, thus affording the desired nucleosyl amino acid building blocks (*S*)-**9** (94% yield) and (*R*)-**9** (90% yield), each in diastereomerically pure form (Scheme 2). Overall, it can be concluded that the omission of the 3-(*N*-BOM) protecting group did not lead to improvements in the synthesis of **9**, but rather made the sequence of Wittig–Horner olefination and asymmetric hydrogenation slightly less efficient. In contrast to uridine derivatives [48], thymidine analogues are significantly more robust towards unwanted reduction of the 5,6-double bond in heterogeneously catalyzed hydrogenolysis reactions with palladium catalysts. Therefore, the absence of the BOM group was not advantageous for the deprotection step following the asymmetric hydrogenation reaction.

**Scheme 3:**
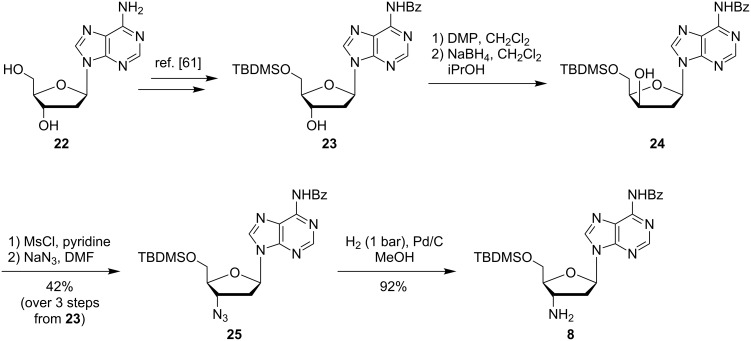
Synthesis of protected 3'-amino-2',3'-dideoxyadenosine **8**.

The second challenge on the way to target phosphoramidites **7** was the synthesis of protected 3'-amino-2',3'-dideoxyadenosine **8** on a sufficient scale. Richert and Eisenhuth have published a comprehensive report on the synthesis of all four 3'-amino-2',3'-dideoxynucleosides with canonical bases [61]. We have decided to mainly follow their strategy for the synthesis of adenosine derivative **8**, though some modifications were applied (Scheme 3). Starting from 2'-deoxyadenosine (**22**), *N*-6-benzoyl-5'-*O*-TBDMS-2'-deoxyadenosine (**23**) was prepared [61] and subjected to oxidation of the 3'-hydroxy functionality to a keto group with Dess–Martin periodinane (DMP). This was followed by reduction with sodium borohydride via nucleophilic attack of the keto group from the sterically less hindered α-face, therefore resulting in the formation of the 3'-*xylo* derivative **24**. A tight control of the reaction conditions, i.e., amounts of reagents, reaction time and temperature, proved to be important for this transformation (see Supporting Information File 1). It was observed that the time period needed for the oxidation step was dependent on the scale of the reaction and that the sensitive keto intermediate decomposed when this time period was unreasonably exceeded. The obtained product **24** still contained minor amounts of aromatic byproducts from the oxidizing agent, which were difficult to remove by column chromatography at this stage. Therefore, this material was employed in subsequent transformations without further attempts to remove the aforementioned impurities. Several methods are known to perform the nucleophilic displacement at the 3'-position of the 3'-*xylo* intermediate with azide as a nucleophile [61–65]. We have found that robust results for this reaction could be achieved by activation of the 3'-hydroxy group as a mesylate, followed by treatment with sodium azide in DMF at elevated temperature (110 °C). This protocol furnished 3'-azido derivative **25** in a moderate, but reliably obtained yield of 42% over 3 steps from **23**. Azido nucleoside **25** was finally reduced to the 3'-amino analogue **8** by standard hydrogenation in 92% yield (Scheme 3).

**Scheme 4:**
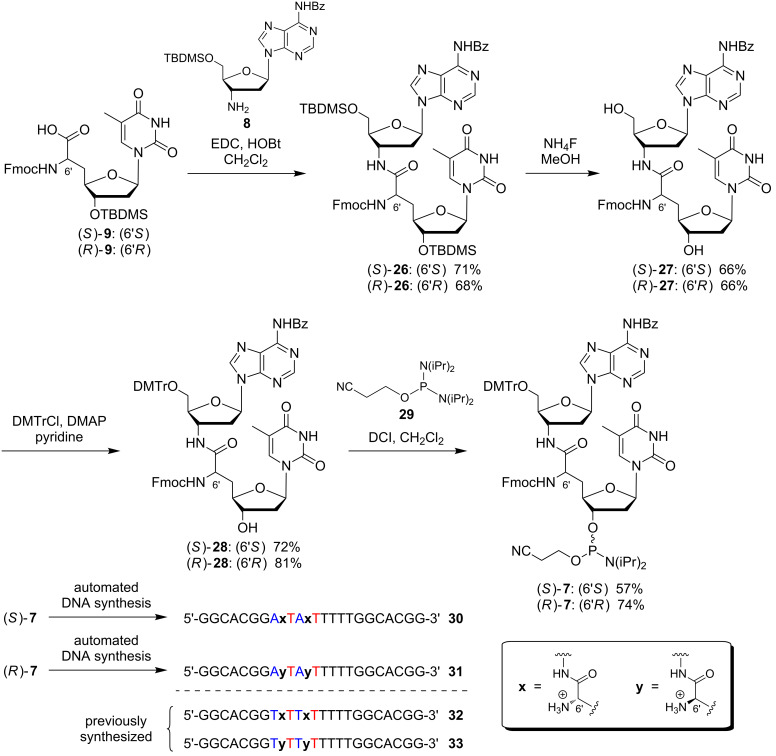
Synthesis of target phosphoramidites (*S*)-**7** and (*R*)-**7** and of two NAA-modified DNA oligonucleotides **30**, **31** (DCI = 4,5-dicyanoimidazole); sites of NAA-modifications in the oligonucleotides are indicated as **x** (6'*S*) and **y** (6'*R*), all other linkages were native phosphates.

With all building blocks **8**, (*S*)-**9** and (*R*)-**9** in hand, the dimeric target structures could be constructed (Scheme 4). Using a standard procedure for peptide coupling, suitably protected nucleosyl amino acids (*S*)-**9** and (*R*)-**9** were activated and reacted with amine **8** to give bis-*O*-silylated NAA-linked A–T dimers (*S*)-**26** and (*R*)-**26** in yields of 71% and 68%, respectively. For the subsequent desilylation reaction, several reaction conditions were tested, among them acidic silyl ether cleavage with hydrochloric acid in methanol or treatment with triethylamine trihydrofluoride (3HF•NEt_3_). However, the only successful method was the conversion of both epimers of **26** with ammonium fluoride in methanol at elevated temperature, which afforded diols (*S*)-**27** and (*R*)-**27**, each in 66% yield. DMTr protection under standard conditions then provided intermediates (*S*)-**28** and (*R*)-**28** in yields of 72% and 81%, respectively. Finally, phosphitylation of the 3'-hydroxy group gave target phosphoramidites (*S*)-**7** (57% yield) and (*R*)-**7** (74% yield), each with defined stereochemistry at the 6'-position. For this reaction, 2-cyanoethyl *N*,*N*,*N′*,*N′*-tetraisopropylphosphordiamidite **29** was used under slightly acidic conditions, i.e., in the presence of the activator 4,5-dicyanoimidazole (DCI, Scheme 4). The alternative method for the introduction of the phosphoramidite functionality, i.e., treatment with the respective chlorophosphite in the presence of a base, resulted in unwanted concomitant cleavage of the Fmoc group.

In order to demonstrate their principle synthetic versatility, 'dimeric' phosphoramidites (*S*)-**7** and (*R*)-**7** were employed for the automated synthesis of two DNA oligonucleotides **30** and **31**, each bearing two NAA-modifications with defined stereochemistry at the NAA-linkage ((6'*S*) or (6'*R*)) and therefore displaying partially zwitterionic backbone structures. After assembly on the synthesizer and base-mediated cleavage from the solid support, the desired full-length products were purified, isolated and identified by ESI mass spectrometry. The sequence of **30** and **31** resembled the sequence of model oligonucleotides **32** and **33** prepared for our initial studies on the NAA-modification [38] (Scheme 4). This strategy will enable systematic future studies on the potential influence of the base sequence on the properties of NAA-modified oligonucleotides.

## Conclusion

In summary, we have successfully accomplished the stereoselective synthesis of 'dimeric' A–T phosphoramidite building blocks (*S*)-**7** and (*R*)-**7** for the preparation of novel NAA-modified DNA oligonucleotides with partially zwitterionic backbone structures. The required nucleosyl amino acid intermediates (*S*)-**9** and (*R*)-**9** were synthesized from thymidine in overall yields of 32% and 31%, respectively, over 9 steps when the nucleobase was 3-(*N*-BOM)-protected [38]. It was possible to slightly shorten this route to 8 steps by leaving the nucleobase unprotected, but the key steps of the synthesis, i.e., Wittig–Horner reaction and subsequent asymmetric hydrogenation, proceeded less efficiently in this case. Coupling of (*S*)-**9** and (*R*)-**9** with protected 3'-amino-2',3'-dideoxyadenosine **8** (obtained from protected 2'-deoxyadenosine derivative **23** in 39% overall yield over 4 steps) and some further transformations furnished target phosphoramidites (*S*)-**7** and (*R*)-**7** in overall yields of 19% and 27%, respectively, over 4 steps. Phosphoramidites (*S*)-**7** and (*R*)-**7** were then employed for solid phase-supported automated DNA synthesis, which afforded novel zwitterionic oligonucleotides **31** and **32** bearing the NAA-modification at A–T sites. Overall, it was therefore demonstrated that the synthesis of different 'dimeric' NAA-linked X–T phosphoramidites with X representing pyrimidine or purine nucleobases appears to be feasible. This will enable the preparation of NAA-modified oligonucleotides with significant variations in the base sequence. We are currently finishing the synthesis of a comprehensive set of corresponding X–T phosphoramidites (including C–T and G–T congeners). It is planned to use these reagents to perform a systematic study on the influence of the base sequence on the properties of NAA-modified oligonucleotides, particularly on duplex stability. This will set the stage for further investigations, particularly on oligonucleotides with fully zwitterionic backbone structures and also on the interaction of NAA-modified oligonucleotides with proteins such as nucleases and polymerases.

## Supporting Information

Supporting Information features preparation, analytical data and copies of ^1^H, ^13^C and ^31^P NMR spectra of compounds **7**, **8**, **11**, **16**, **18**, **20**, **21**, **24**–**28** as well as preparation and analytical data of oligonucleotides **30** and **31**.

File 1 Experimental procedures and NMR spectra of compounds **7**, **8**, **11**, **16**, **18**, **20**, **21**, **24**–**28**, **30**, and **31**.
