# Supplementary material for: NAA-modified DNA oligonucleotides with zwitterionic backbones: stereoselective synthesis of A–T phosphoramidite building blocks
Source: Beilstein J Org Chem. 2015 Jan 13;11:50–60. doi: 10.3762/bjoc.11.8 (PMC4311628; doi:10.3762/bjoc.11.8)
Supplement: File 1 — Experimental procedures and NMR spectra of compounds 7, 8, 11, 16, 18, 20, 21, 24–28, 30, and 31. [file Beilstein_J_Org_Chem-11-50-s001.pdf]

Supporting Information

for

NAA-modified DNA oligonucleotides with zwitterionic backbones: stereoselective synthesis of A–T phosphoramidite building blocks

Boris Schmidtgall[1,2], Claudia Höbartner[3,4] and Christian Ducho*[1,2]

Address: [1]Department of Chemistry, University of Paderborn, Warburger Str. 100, 33 098 Paderborn, Germany, [2]Department of Pharmacy, Pharmaceutical and Medicinal Chemistry, Saarland University, Campus C2 3, 66 123 Saarbrücken, Germany, [3]Max-Planck-Institute for Biophysical Chemistry, Am Fassberg 11, 37 077 Göttingen, Germany and [4]Department of Chemistry, Institute of Organic and Biomolecular Chemistry, Georg-August-University Göttingen, Tammannstr. 2, 37 077 Göttingen, Germany

Email: Christian Ducho - christian.ducho@uni-saarland.de

* Corresponding author

Experimental procedures and NMR spectra of compounds 7, 8, 11, 16, 18, 20, 21, 24–28, 30, and 31

Table of contents

Synthesis of phosphoramidites (*S*)-**7** and (*R*)-**7**………...……….. S2

^{1}H, ^{13}C and ^{31}P NMR spectra of synthesized compounds...…..………………..…........... S25

Oligonucleotide synthesis and analytical data of oligonucleotides.................................. S40

References.........................…………………………………………………….................. S43

Synthesis of phosphoramidites (*S*)-7 and (*R*)-7

General methods. The synthesis of the following compounds has been published before in the Supplementary Information of our first Communication on NAA-modified oligonucleotides [S1]: (*S*)-**9**, (*R*)-**9**, **10**, **14**, **15**, *Z*-**17**, *E*-**17**, (*S*)-**19**, (*R*)-**19**, (*S*)-**21**, (*R*)-**21**. Compounds **12** [S2-S5] and **23** [S6] were prepared according to established procedures. All other chemicals were purchased from standard suppliers. Reactions involving oxygen and/or moisture sensitive reagents were carried out under an atmosphere of argon using anhydrous solvents. Anhydrous solvents were obtained in the following manner: THF was dried over sodium/benzophenone and distilled, CH_2Cl_2 was dried over CaH_2 and distilled, MeOH was dried over activated molecular sieves (3 Å) and degassed, MeCN was dried over P_2O_5 and distilled, pyridine was dried over CaH_2 and distilled, toluene was dried over sodium/benzophenone and distilled. The thus obtained solvents were stored over molecular sieves (4 Å, in case of MeOH and MeCN 3 Å). All other solvents were of technical quality and distilled prior to their use, and deionized water was used throughout. Column chromatography was carried out on silica gel 60 (0.040–0.063 mm, 230–400 mesh ASTM, VWR) under flash conditions except where indicated. TLC was performed on aluminum plates precoated with silica gel 60 F_{254} (VWR). Visualization of the spots was carried out using UV light (254 nm) and/or staining under heating (H_2SO_4 staining solution: 4 g vanillin, 25 mL conc. H_2SO_4, 80 mL AcOH and 680 mL MeOH; $KMnO_4$ staining solution: 1 g $KMnO_4$, 6 g K_2CO_3 and 1.5 mL 1.25 M NaOH solution, all dissolved in 100 mL H_2O; ninhydrin staining solution: 0.3 g ninhydrin, 3 mL AcOH and 100 mL 1-butanol). 300 MHz- and 500 MHz-^{1}H and 75 MHz- and 126 MHz-^{13}C as well as 121 MHz-^{31}P NMR spectra were recorded on Varian MERCURY 300, UNITY 300 and INOVA 500 spectrometers. All ^{13}C NMR spectra are H-decoupled. All spectra were recorded at room temperature except where

indicated otherwise and were referenced internally to solvent reference frequencies. For the calibration of ^{31}P NMR signals, 85% phosphoric acid was used as an external standard. Chemical shifts (δ) are quoted in ppm, and coupling constants (*J*) are reported in Hz. Assignment of signals was carried out using H,H-COSY, HSQC and TOCSY spectra obtained on the spectrometers mentioned above. Mass spectra of small molecules were measured on a Finnigan LCQ ion-trap mass spectrometer or on a Bruker microTOF spectrometer. For ESI measurements in the negative mode, solutions of the compounds in pure MeOH were used whereas for measurements in the positive mode, solutions with the addition of 0.1% formic acid were used. High resolution spectra were measured on a Bruker 7 Tesla fourier transform ion cyclotron resonance (FTICR) mass spectrometer. Melting points (mp) were measured on a Büchi instrument and are not corrected. Optical rotations were recorded on a Perkin-Elmer polarimeter 241 with a Na source using a 10 cm cell. Solutions of the compounds (~ 10 mg) in $CHCl_3$ or pyridine (1 mL) were used, and concentrations are given in g/100 mL. Infrared spectroscopy (IR) was performed on a Bruker Vector 22 FTIR spectrometer using a KBr pellet or on a Jasco FT/IR-4100 spectrometer equipped with an integrated ATR unit (GladiATR™, PIKE Technologies) or on a Bruker Vertex 70 spectrometer equipped with a platinum ATR unit. For each compound the wavenumbers (ν) of the nine most intense absorption bands are given in cm^{-1}. UV spectroscopy of small molecules was carried out on a Perkin-Elmer Lambda 2 spectrometer. Measurements were performed with solutions of ~0.1 mg of the compound in 10 mL MeCN and in the range of 190–500 nm. Wavelengths of maximum absorption (λ_{max}) are reported in nm with the corresponding logarithmic molar extinction coefficient given in parenthesis (log ε, ε/dm^3 mol^{-1} cm^{-1}).

(6'*S*)-NAA-modified 2'-deoxyadenosine-thymidine dimer β-cyanoethyl-*N*,*N*-diisopropyl-phosphoramidite ((*S*)-7)

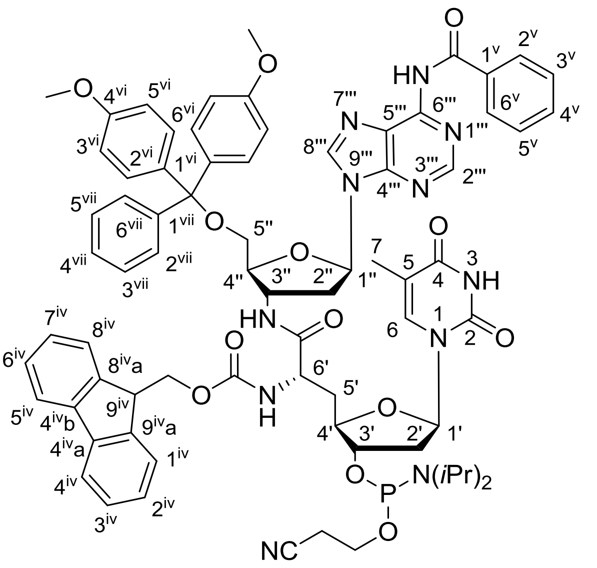

(*S*)-**7**

O-DMTr-protected dimer (*S*)-**28** (210 mg, 0.181 mmol) was coevaporated with pyridine (1 x 2 mL), toluene (1 x 2 mL), and MeCN (1 x 2 mL) and then dissolved in CH_2Cl_2 (2 mL). To this solution, 4,5-dicyanoimidazole (DCI, 21 mg, 0.182 mmol) and a solution of 2-cyanoethyl *N*,*N*,*N'*,*N'*-tetraisopropyl phosphordiamidite (67 mg, 0.219 mmol) in CH_2Cl_2 (1.8 mL) were added. After stirring at room temperature for 1 h, the mixture was diluted with CH_2Cl_2 (40 mL) and washed with sat. $NaHCO_3$ (20 mL). The aqueous layer was re-extracted with CH_2Cl_2 (10 mL). The combined organics were dried over Na_2SO_4 and the solvent was removed under reduced pressure. The resultant crude product was purified by column chromatography (CH_2Cl_2-MeOH 15:1 + 0.5% pyridine). Fractions containing the product were pooled and the solvent was removed under reduced pressure. The thus obtained material was dissolved in CH_2Cl_2 (3.5 mL). This solution was slowly added to hexanes (34 mL) at -20 °C. The resultant fine precipitate was filtered off and dried under reduced pressure yielding 140 mg (0.103 mmol, 57%) of (*S*)-**7** as a colorless powder. TLC R_f 0.25 (CH_2Cl_2-MeOH 95:5). ^{31}P NMR (121 MHz, pyridine-d_5): 149.01, 149.45; MS (ESI$^+$) *m/z* calcd for $C_{74}H_{78}N_{11}NaO_{13}P$ 1382.5416 (M+Na$^+$), found 1382.5378.

(6'*R*)-NAA-modified 2'-deoxyadenosine-thymidine dimer β-cyanoethyl-*N*,*N*-diisopropyl-phosphoramidite ((*R*)-7)

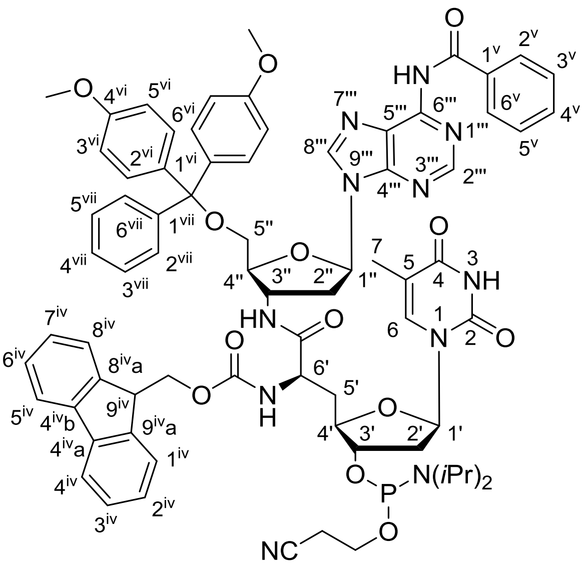

(*R*)-**7**

The synthesis of (*R*)-**7** was performed according to the protocol for the synthesis of (*S*)-**7** with *O*-DMTr-protected dimer (*R*)-**28** (186 mg, 0.160 mmol), 2-cyanoethyl *N*,*N*,*N'*,*N'*-tetraisopropyl phosphordiamidite (58 mg, 0.192 mmol), 4,5-dicyanoimidazole (DCI, 19 mg, 0.16 mmol) and CH_2Cl_2 (1.6 mL) to yield 160 mg (0.118 mmol, 74%) of (*R*)-**7** as a colorless powder. TLC R_f 0.25 (CH_2Cl_2-MeOH 95:5); ^{31}P NMR (121 MHz, pyridine-d_5): 147.34, 147.47; MS (ESI^+) *m/z* calcd for $C_{74}H_{78}N_{11}NaO_{13}P$ 1382.5416 ($M+Na^+$), found 1382.5375.

3'-Amino-6-*N*-benzoyl-5'-*O*-(*tert*-butyldimethylsilyl)-2',3'-dideoxyadenosine (8)

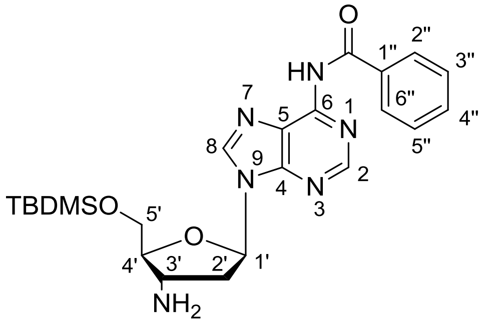

8

To a solution of azide **25** (1.66 g, 3.37 mmol) in MeOH (110 mL), Pd (10% on charcoal, 35 mg, 34 µmol Pd) was added and the resultant suspension was stirred under a hydrogen atmosphere (1 bar) at room temperature for 20 h. The catalyst was removed by filtration through a celite pad. The solvent of the filtrate was removed under reduced pressure yielding 1.45 g (3.09 mmol, 92%) of **8** as a yellowish foam. TLC R_f 0.41 (CH_2Cl_2-MeOH 9:1); mp 70 °C; ^{1}H NMR (300 MHz, CD_3OD): 0.09 (s, 3H, $SiCH_3$), 0.10 (s, 3H, $SiCH_3$), 0.92 (s, 9H, $SiC(CH_3)_3$), 2.50 (ddd, 1H, J = 13.6, 7.4, 7.4 Hz, 2'-H_a), 2.83 (ddd, 1H, J = 13.6, 6.7, 3.5 Hz, 2'-H_b), 3.84-3.91 (m, 2H, 3'-H, 4'-H), 3.93 (dd, 1H, J = 11.3, 3.4 Hz, 5'-H_a), 4.01 (dd, 1H, J = 11.3, 3.0 Hz, 5'-H_b), 6.54 (dd, 1H, J = 7.4, 3.5 Hz, 1'-H), 7.58 (dd, 2H, J = 7.5, 7.5 Hz, 3''-H, 5''-H), 7.67 (t, 1H, J = 7.5 Hz, 4''-H), 8.02 (d, 2H, J = 8.3 Hz, 2''-H, 6''-H), 8.68 (s, 1H, 2-H), 8.72 (s, 1H, 8-H); ^{13}C NMR (75 MHz, CD_3OD): -6.7, 17.9, 25.1, 38.1, 60.5, 62.8, 84.5, 85.1, 123.4, 127.9, 128.9, 132.8, 133.8, 141.4, 149.6, 151.2, 152.7, 164.6; IR (ATR) ν 2926, 1685, 1609, 1580, 1447, 1250, 1070, 835, 779, 707; UV (MeCN) λ_{max} (log ε) 223 (3.86), 259 (3.48); $[\alpha]_D^{20}$ -15.0 (c 1.0, $CHCl_3$); MS (ESI^+) *m/z* calcd for $C_{23}H_{33}N_6O_3Si$ 469.2378 ($M+H^+$), found 469.2374.

3'-*O*-(*tert*-Butyldimethylsilyl)thymidine-5'-aldehyde (11)

To a solution of 3'-*O*-(*tert*-butyldimethylsilyl)thymidine **16** (3.46 g, 9.71 mmol) in MeCN (90 mL), IBX (6.80 g 24.3 mmol) was added and the resultant suspension was heated under reflux for 30 min. After cooling to room temperature, the suspension was filtered. The insoluble residue was washed with EtOAc (3 x 30 mL) and the solvent of the combined filtrates was removed under reduced pressure to yield 3.44 g (9.70 mmol, quant.) of **11** as a colourless foam which was used in the subsequent reaction without further purification.

3'-*O*-(*tert*-Butyldimethylsilyl)thymidine (16)

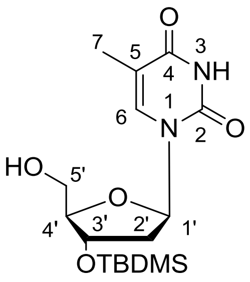

16

A solution of 3',5'-di-*O*-(*tert*-butyldimethylsilyl)thymidine **13** (7.00 g, 14.9 mmol, prepared from thymidine with TBDMS chloride and imidazole in pyridine as solvent in quantitative yield) in dry MeOH was treated with acetyl chloride (291 mg, 265 µL, 3.73 mmol) at -10 °C. The resultant solution was slowly warmed to 0 °C and then stirred at 0 °C for 3 h. The reaction was carefully monitored by TLC (petroleum ether-EtOAc 1:1). It was quenched by the addition of sat. $NaHCO_3$ solution (50 mL) and further stirring for 15 min. The obtained mixture was diluted with EtOAc (300 mL). The organic layer was washed with sat. $NaHCO_3$ solution (2 x 100 mL), water (100 mL) and brine (100 mL) and dried over Na_2SO_4. The solvent was removed under reduced pressure. The resultant oily crude product was purified by column chromatography (petroleum ether-EtOAc 1:1) yielding 3.50 g (9.82 mmol, 66%) of **16** as a white powder. TLC R_f 0.15 (petroleum ether-EtOAc 1:1); mp 109 °C; ^{1}H NMR (300 MHz, $CDCl_3$): 0.05 (s, 6H, $SiCH_3$), 0.86 (s, 9H, $SiC(CH_3)_3$), 1.86 (d, 3H, J = 1.1 Hz, 7-H), 2.16 (ddd, 1H, J = 13.4, 6.4, 3.6 Hz, 2'-H_a), 2.31 (ddd, 1H, J = 13.4, 6.7,6.7 Hz, 2'-H_b), 2.96 (brs, 1H, OH), 3.72 (dd, 1H, J = 12.2, 3.3 Hz, 5'-H_a), 3.84-3.91 (m, 2H, 4'-H, 5'-H_b), 4.46 (ddd, 1H, J = 6.4, 3.6, 3.6 Hz, 3'-H), 6.12 (dd, 1H, J = 6.7, 6.7 Hz, 1'-H), 7.37 (d, 1H, J = 1.1 Hz, 6-H); ^{13}C NMR (75 MHz, $CDCl_3$): -4.9, -4.7, 12.4, 17.9, 25.7, 40.5, 61.9, 71.5, 86.7, 87.6, 110.9, 137.0, 150.4, 164.0; IR (KBr) ν 1690, 1666, 1108, 1088, 1056, 1032, 1017, 831, 774; UV (MeCN) λ_{max} (log ε) 207 (3.99), 265 (4.02); $[\alpha]_D^{20}$ +27.0 (c 1.0, $CHCl_3$); MS (ESI^+) *m/z* calcd for $C_{16}H_{28}N_2NaO_5Si$ 379.1660 ($M+Na^+$), found 379.1659.

Didehydro thymidinyl amino acid ester *Z*-18 and didehydro thymidinyl amino acid ester *E*-18

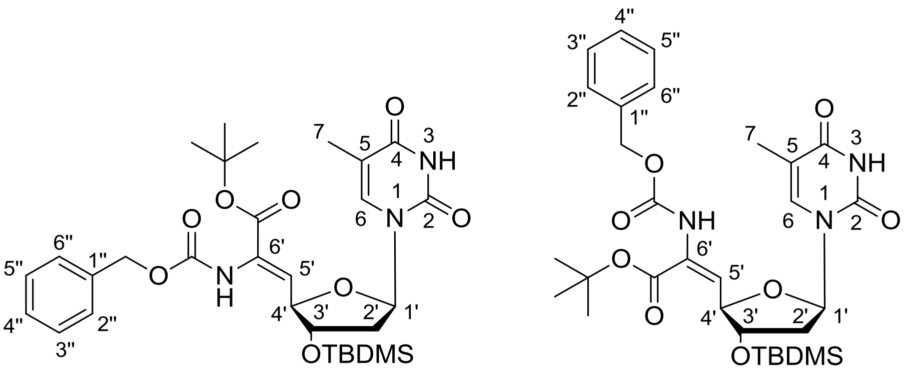

Z-**18** *E*-**18**

To a solution of KO*t*-Bu (1.12 g, 9.96 mmol) in THF (95 mL), a solution of phosphonate **12** (3.95 g, 10.6 mmol) [S2-S5] in THF (75 mL) was added dropwise at -78 °C. After stirring at -78 °C for 5 min, a solution of 3'-*O*-(*tert*-butyldimethylsilyl)thymidine-5'-aldehyde **11** (3.44 g, 9.70 mmol) in THF (40 mL) was added slowly to the reaction mixture maintaining the temperature below -70 °C. The resultant solution was stirred for 16 h and allowed to slowly warm to room temperature during this time. MeOH (5 mL) and EtOAc (300 mL) were then added at 0 °C. The mixture was washed with water (3 x 100 mL), dried over Na_2SO_4 and the solvent was removed under reduced pressure. The resultant viscous crude product was purified by column chromatography (petroleum ether-EtOAc 3:2) yielding 3.83 g (6.36 mmol, 66%) of a mixture of *Z*-**18** and *E*-**18** (*Z*:*E* = 91:9). Analytical amounts of pure isomers *Z*-**18** and *E*-**18** were obtained by repeated column chromatography under the aformentioned conditions. *Z*-**18**: TLC R_f 0.33 (petroleum ether-EtOAc 3:2); mp 79 °C; ^{1}H NMR (300 MHz, $CDCl_3$): 0.06 (s, 6H, $SiCH_3$), 0.86 (s, 9H, $SiC(CH_3)_3$), 1.45 (s, 9H, $OC(CH_3)_3$), 1.89 (d, 3H, J = 1.1 Hz, 7-H), 2.13 (ddd, 1H, J = 13.6, 6.5, 6.1 Hz, 2'-H_a), 2.33 (ddd, 1H, J = 13.4, 6.5, 4.4 Hz, 2'-H_b), 4.33 (ddd, 1H, J = 6.1, 4.4, 4.4 Hz, 3'-H), 4.67 (dd, 1H, J = 7.8, 4.4 Hz, 4'-H), 5.09 (d, 1H, J = 12.1 Hz, $BnCH_2$-H_a), 5.13 (d, 1H, J = 12.1 Hz, $BnCH_2$-H_b), 6.13 (d, 1H, J = 7.8 Hz, 5'-H), 6.15 (dd, 1H, J = 6.7, 6.7 Hz, 1'-H), 6.88 (brs, 1H, NH), 7.08 (d, 1H,

J = 1.1 Hz, 6-H), 7.33-7.37 (m, 3H, aryl-H), 7.50-7.55 (m, 1H, aryl-H), 7.68-7.74 (m, 1H, aryl-H); ^{13}C NMR (75 MHz, $CDCl_3$): -5.0, -4.9, 12.6, 17.8, 25.6, 27.8, 40.4, 67.5, 75.9, 82.5, 82.6, 86.1, 111.0, 124.9, 128.2, 128.3, 128.5, 130.5, 135.4, 135.7, 150.1, 153.6, 162.7, 163.8; IR (KBr) ν 1686, 1465, 1256, 1223, 1151, 1046, 831, 774, 693; UV (MeCN) λ_{max} (log ε) 204 (4.40), 259 (4.15); $[\alpha]_D^{20}$ +69.9 (c 1.1, $CHCl_3$); MS (ESI^+) *m/z* calcd for $C_{30}H_{43}N_3NaO_8Si$ 624.2717 ($M+Na^+$), found 624.2705. *E*-**2**: ^{1}H NMR (300 MHz, $CDCl_3$): 0.05 (s, 6H, $SiCH_3$), 0.87 (s, 9H, $SiC(CH_3)_3$), 1.52 (s, 9H, $OC(CH_3)_3$), 1.90 (s, 3H, 7-H), 2.08 (ddd, 1H, J = 13.5, 7.7, 5.6 Hz, 2'-H_a), 2.33 (ddd, 1H, J = 13.5, 6.1, 2.6 Hz, 2'-H_b), 4.21 (ddd, 1H, J = 5.3, 2.6, 2.6 Hz, 3'-H), 5.10 (d, 1H, J = 12.3 Hz, $BnCH_2$-H_a), 5.15 (d, 1H, J = 12.3 Hz, $BnCH_2$-H_b), 5.36 (dd, 1H, J = 9.8, 2.5 Hz, 4'-H), 6.31 (dd, 1H, J = 7.7, 6.1 Hz, 1'-H), 6.76 (d, 1H, J = 9.8 Hz, 5'-H), 7.11 (s, 1H, Cbz-NH), 7.18-7.39 (m, 5H, aryl-H), 7.28 (s, 1H, 6-H); ^{13}C NMR (75 MHz, $CDCl_3$): -4.7, -4.7, 12.5, 18.0, 25.7, 28.0, 40.6, 66.9, 77.0, 83.1, 84.4, 85.6, 111.1, 120.2, 128.0, 128.0, 128.4, 128.8, 135.4, 136.1, 150.3, 153.1, 161.6, 163.8.

_N_-Cbz-Protected (6'_S_)-thymidinyl amino acid ester (_S_)-20

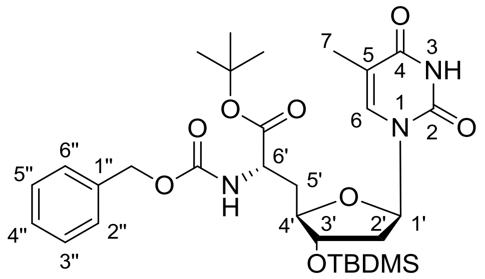

(*S*)-**20**

The reaction was performed under strict exclusion of oxygen. Oxygen was removed from a solution of didehydro thymidinyl amino acid ester **18** (mixture of *Z*- and *E*-isomers (91:9), 1.80 g, 2.99 mmol) in MeOH (100 mL) by a steady stream of argon over 15 min. (*S*,*S*)-Me-DuPHOS-Rh (100 mg, 165 µmol) was then added and the solution was stirred under a hydrogen atmosphere (1 bar) for 9 d. The solvent was removed under reduced pressure. The

resultant crude product was purified by column chromatography (petroleum ether-EtOAc 3:2) yielding 1.67 g (2.77 mmol, 93%) of (*S*)-**20** as a colorless oil. TLC R_f 0.45 (petroleum ether-EtOAc 3:2); ^{1}H NMR (300 MHz, $CDCl_3$): 0.05 (s, 6H, $SiCH_3$), 0.87 (s, 9H, $SiC(CH_3)_3$), 1.43 (s, 9H, $OC(CH_3)_3$), 1.97 (s, 3H, 7-H), 2.00-2.10 (m, 2H, 2'-H_a, 5'-H_a), 2.13-2.27 (m, 2H, 2'-H_b, 5'-H_b), 3.90-3.99 (m, 1H, 3'-H), 4.06 (ddd, 1H, *J* = 6.6, 4.4, 4.4 Hz, 4'-H), 4.42 (ddd, 1H, *J* = 7.1, 4.7, 4.7 Hz, 6'-H), 5.10 (s, 2H, $BnCH_2$), 5.54 (d, 1H, *J* = 7.1 Hz, 6'-NH), 6.18 (dd, 1H, *J* = 6.5, 6.5 Hz, 1'-H), 7.29-7.40 (m, 5H, aryl-H), 7.37 (s, 1H, 6-H), 8.34 (brs, 1H, 3-NH); ^{13}C NMR (75 MHz, $CDCl_3$): -4.9, -4.7, 12.3, 17.9, 25.6, 27.9, 36.0, 40.2, 52.0, 66.8, 74.9, 82.6, 83.0, 85.1, 111.1, 128.0, 128.1, 128.5, 135.8, 136.2, 150.2, 155.5, 163.9, 170.5; IR (KBr) ν 1686, 1466, 1366, 1251, 1151, 1093, 1045, 835, 774; UV (MeCN) λ_{max} (log ε) 205 (4.66), 264 (4.40); $[\alpha]_D^{20}$ +58.4 (c 1.2, $CHCl_3$); MS (ESI^+) *m/z* calcd for $C_{30}H_{45}N_3NaO_8Si$ 626.2868 ($M+Na^+$), found 626.2865.

***N*-Cbz-Protected (6'*R*)-thymidinyl amino acid ester (*R*)-20**

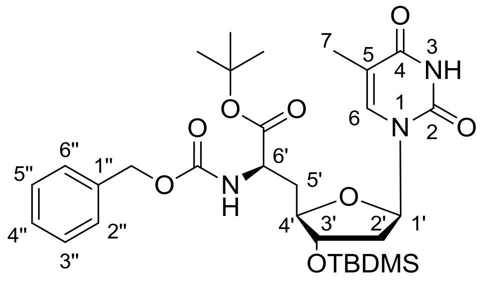

(*R*)-**20**

The synthesis of (*R*)-**20** was performed according to the protocol for the synthesis of (*S*)-**20** with didehydro thymidinyl amino acid ester **18** (mixture of *Z*- and *E*-isomers (91:9), 920 mg, 1.53 mmol), (*R*,*R*)-Me-DuPHOS-Rh (60 mg, 96 µmol), MeOH (40 mL) and a reaction time of 21 d to yield 710 mg (1.18 mmol, 77%) of (*R*)-**20** as a colorless foam. TLC R_f 0.42 (petroleum ether-EtOAc 3:2); mp 76 °C; ^{1}H NMR (300 MHz, $CDCl_3$): 0.04 (s, 3H, $SiCH_3$), 0.04 (s, 3H, $SiCH_3$) 0.85 (s, 9H, $SiC(CH_3)_3$), 1.43 (s, 9H, $OC(CH_3)_3$), 1.85 (s, 3H, 7-H), 2.01

(ddd, 1H, J = 14.3, 10.3, 4.3 Hz, 2'-H_a), 2.09 (ddd, 1H, J = 13.4, 6.8, 6.8 Hz, 5'-H_a), 2.14-2.23 (m, 2H, 2'-H_b, 5'-H_b), 3.78 (ddd, 1H, J = 10.3, 4.9, 2.4 Hz, 3'-H), 4.06 (ddd, 1H, J = 6.8, 4.9, 4.9 Hz, 4'-H), 4.40-4.45 (m, 1H, 6'-H), 5.10 (s, 2H, $BnCH_2$), 5.63 (d, 1H, J = 8.6 Hz, 6'-NH), 6.08 (dd, 1H, J = 6.4, 6.4 Hz, 1'-H), 7.07 (s, 1H, 6-H), 7.24-7.32 (m, 5H, aryl-H), 9.34 (brs, 1H, 3-NH); ^{13}C NMR (75 MHz, $CDCl_3$): -4.9, -4.7, 12.5, 17.8, 25.6, 27.9, 35.0, 40.1, 52.6, 66.8, 74.8, 82.4, 83.3, 85.5, 111.0, 127.9, 128.1, 128.4, 135.4, 136.2, 150.0, 155.9, 163.8, 170.6; IR (KBr) ν 1690, 1466, 1361, 1250, 1151, 1098, 1055, 831, 779; UV (MeCN) λ_{max} (log ε) 202 (4.31), 264 (4.01); $[\alpha]_D^{20}$ +62.3 (c 1.0, $CHCl_3$); MS (ESI$^-$) *m/z* calcd for $C_{30}H_{44}N_3O_8Si$ 602.2903 (M-H^+), found 602.2903.

***N*-Fmoc-Protected (6'*S*)-thymidinyl amino acid ester (*S*)-21**

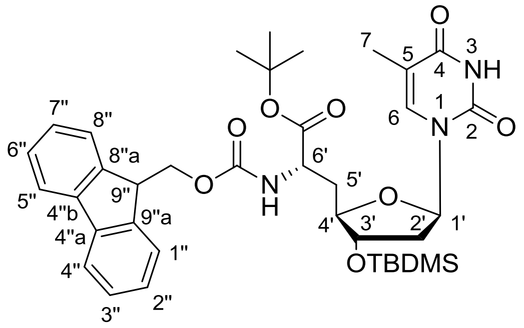

(*S*)-**21**

From (*S*)-**19**: as published before [S1]. From (*S*)-**20**: To a solution of *N*-Cbz-protected (6'*S*)-thymidinyl amino acid ester (*S*)-**20** (1.51 g, 2.50 mmol) in MeOH (45 mL), Pd (10% on charcoal, 80 mg, 75 µmol Pd) was added and the resultant suspension was stirred under a hydrogen atmosphere (1 bar) at room temperature for 1 h. The catalyst was removed by filtration through a celite pad, and the solvent of the filtrate was removed under reduced pressure. The resultant oily crude product (1.16 g) was dissolved in THF (19 mL). To this solution, NEt_3 (0.85 mL, 0.62 g, 6.2 mmol) and FmocCl (798 mg, 3.09 mmol) were added at 0 °C. After stirring at 0 °C for 1 h, water (1.5 mL) was added, and the reaction mixture was poured into EtOAc (50 mL). The organic layer was washed with water (2 x 20 mL) and brine

(20 mL) and dried over Na_2SO_4. The solvent was removed under reduced pressure. The resultant crude product was purified by column chromatography (petroleum ether-EtOAc 7:3) yielding 1.45 g (2.10 mmol, 84% over 2 steps from (*S*)-**20**) of (*S*)-**21** as a colorless foam. Analytical data were found to be identical to those reported before [S1].

N-Fmoc-Protected (6'*R*)-thymidinyl amino acid ester ((*R*)-21)

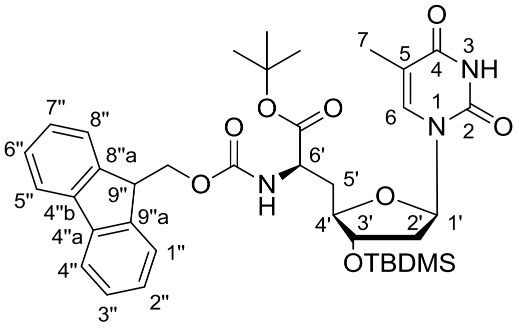

(*R*)-**21**

From (*R*)-**19**: as published before [S1]. From (*R*)-**20**: The synthesis of (*R*)-**21** was performed according to the protocol for the synthesis of (*S*)-**21** with *N*-Cbz-protected (6'*R*)-thymidinyl amino acid ester (*R*)-**20** (1.50 g, 2.48 mmol), Pd (10% on charcoal, 80 mg, 75 µmol Pd), MeOH (45 mL), 1.06 g resultant crude product from the first reaction step, FmocCl (798 mg, 3.09 mmol), NEt_3 (0.85 mL, 0.62 g, 6.2 mmol) and THF (19 mL) to yield 1.34 g (1.94 mmol, 78% over 2 steps from (*R*)-**20**) of (*R*)-**21** as a colorless foam. Analytical data were found to be identical to those reported before [S1].

6-*N*-Benzoyl-5'-*O*-(*tert*-butyldimethylsilyl)-2'-deoxy-3'-*xylo*-adenosine (24)

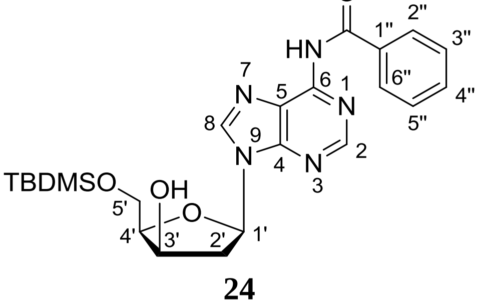

24

To a solution of Dess-Martin periodinane (DMP, 2.70 g, 6.36 mmol), 6-*N*-benzoyl-5'-*O*-(*tert*-butyldimethylsilyl)-2'-deoxyadenosine (**23**, 2.00 g, 4.26 mmol) [S6] was added at 0 °C. After stirring at 0 °C for 30 min, the reaction mixture was allowed to warm to room temperature and was stirred at room temperature for 14 h. After addition of iPrOH (40 mL), the reaction mixture was cooled to -60 °C. Freshly powdered $NaBH_4$ (320 mg, 8.46 mmol) was added and the resultant solution was stirred at -60 °C for 16 h. The reaction was quenched by the addition of acetone (40 mL) at 60 °C and then diluted with EtOAc (300 mL). The solution was washed with sat. $NaHCO_3$ solution (2 x 100 mL) and brine (100 mL). The combined aqueous layers were re-extracted with EtOAc (100 mL). The combined organics were dried over Na_2SO_4 and the solvent was removed under reduced pressure. The resultant crude product was purified by column chromatography (CH_2Cl_2-MeOH 97:3) yielding 1.43 g of impure **24** (containing some aromatic byproducts from the oxidant) as a colorless foam. TLC R_f 0.30 (CH_2Cl_2-MeOH 95:5); mp 80 °C; ^{1}H NMR (300 MHz, $CDCl_3$): 0.04 (s, 3H, $SiCH_3$), 0.06 (s, 3H, $SiCH_3$), 0.87 (s, 9H, $SiC(CH_3)_3$), 2.56 (dd, 1H, J = 15.3, 2.5 Hz, 2'-H_a), 2.86 (ddd, 1H, J = 15.3, 9.1, 6.0 Hz, 2'-H_b), 3.97-4.03 (m, 2H, 4'-H, 5'-H_a), 4.05-4.12 (m, 1H, 5'-H_b), 4.51-4.57 (m, 1H, 3'-H), 5.72-5.80 (m, 1H, 3'-OH), 6.30 (dd, 1H, J = 9.1, 2.5 Hz, 1'-H), 7.51 (dd, 2H, J = 7.4, 7.4 Hz, 3''-H, 5''-H), 7.59 (t, 1H, J = 7.4 Hz, 4''-H), 8.00 (d, 2H, J = 7.4 Hz, 2''-H, 6''-H), 8.33 (s, 1H, 2-H), 8.77 (s, 1H, 8-H), 9.09 (s, 1H, 6-NH); ^{13}C NMR (75 MHz, $CDCl_3$): -5.4, 18.3, 25.9, 41.0, 62.1, 71.1, 84.2, 84.8, 123.8, 127.9, 128.9, 132.9,133.6, 133.6, 143.1, 149.9, 152.1, 164.5; IR (ATR) ν 2926, 1695, 1609, 1580, 1452, 1250, 1065, 832, 779, 702; UV (MeCN) λ_{max} (log ε) 228 (4.11), 281 (4.16); $[\alpha]_D^{20}$ -28.0 (c 1.0, $CHCl_3$); MS (ESI^+) *m/z* calcd for $C_{23}H_{32}N_5O_4Si$ 470.2218 ($M+H^+$), found 470.2217.

3'-Azido-6-*N*-benzoyl-5'-*O*-(*tert*-butyldimethylsilyl)-2',3'-dideoxyadenosine (25)

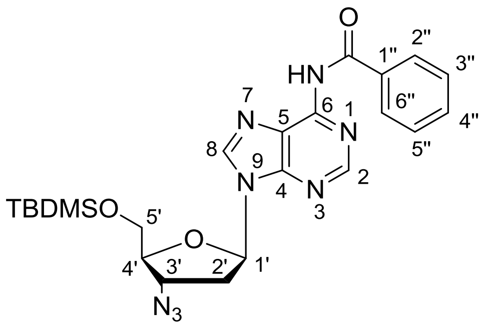

25

To a solution of impure 6-*N*-benzoyl-5'-*O*-(*tert*-butyldimethylsilyl)-2'-deoxy-3'-*xylo*-adenosine (**24**, 500 mg, containing some aromatic byproducts from the previous oxidation reaction) in pyridine (8 mL), methanesulfonyl chloride (410 µL, 610 mg, 5.30 mmol) was slowly added at 0 °C. The reaction mixture was allowed to warm to room temperature and then stirred at room temperature for 4 h. The solution was diluted with CH_2Cl_2 (100 mL), washed with sat. $NaHCO_3$ solution (50 mL) and water (50 mL) and dried over Na_2SO_4. The solvent was removed under reduced pressure. The resultant viscous crude product was coevaporated with toluene (2 x 4 mL) and CH_2Cl_2 (10 mL) yielding 580 mg of the mesylate as a viscous brown oil that was dissolved in DMF (16 mL). To this solution, NaN_3 (640 mg, 9.84 mmol) was added, and the resultant suspension was stirred at 110 °C for 1.5 h. After cooling to room temperature, the reaction mixture was diluted with EtOAc (100 mL). The solution was washed with sat. $NaHCO_3$ solution (2 x 50 mL), water (2 x 50 mL) and brine (50 mL) and was dried over Na_2SO_4. The solvent was removed under reduced pressure. The resultant crude product was purified by column chromatography (CH_2Cl_2-MeOH 98:2) yielding 313 mg (0.633 mmol, 42% over 3 steps from **23**) of **25** as a viscous yellow oil. TLC R_f 0.29 (*iso*-hexanes-EtOAc 3:2); ^{1}H NMR (300 MHz, $CDCl_3$): 0.09 (s, 3H, $SiCH_3$), 0.10 (s, 3H, $SiCH_3$), 0.91 (s, 9H, $SiC(CH_3)_3$), 2.61 (ddd, 1H, J = 13.6, 6.6, 5.8 Hz, 2'-H_a), 2.91 (ddd, 1H, J = 13.6, 6.6, 5.8 Hz, 2'-H_b), 3.84 (dd,1H, J = 11.3, 3.3 Hz, 5'-H_a), 3.95 (dd, 1H, J = 11.3, 4.1 Hz, 5'-H_b), 4.07-4.11 (m, 1H, 4'-H), 4.49 (ddd, 1H, J = 6.6, 6.6, 5.3 Hz, 3'-H), 6.43 (dd,

1H, *J* = 5.8, 5.8 Hz, 1'-H), 7.52 (dd, 2H, *J* = 7.5, 7.5 Hz, 3"-H, 5"-H), 7.60 (t, 1H, *J* = 7.5 Hz, 4"-H), 8.02 (d, 2H, *J* = 7.5 Hz, 2"-H, 6"-H), 8.30 (s, 1H, 2-H), 8.79 (s, 1H, 8-H), 9.05 (brs, 1H, 6-NH); ^{13}C NMR (75 MHz, $CDCl_3$): -5.5, -5.4, 18.4, 25.9, 38.1, 60.5, 62.8, 84.5, 85.1, 123.4, 127.9, 128.9, 132.8, 133.8, 141.4, 149.6, 151.2, 152.7, 164.6; IR (ATR) ν 2926, 2100, 1695, 1609, 1452, 1247, 1070, 835, 774, 702; UV (MeCN) λ_{max} (log ε) 279 (4.43); $[\alpha]_D^{20}$ -9.0 (c 1.0, $CHCl_3$); MS (ESI$^+$) *m/z* calcd for $C_{23}H_{31}N_8O_3Si$ 495.2283 (M+H$^+$), found 495.2284.

Bis-*O*-TBDMS-protected (6'*S*)-thymidinyl amino acid (3'-amino-2',3'-dideoxy-3'-yl)-amide (*S*)-26

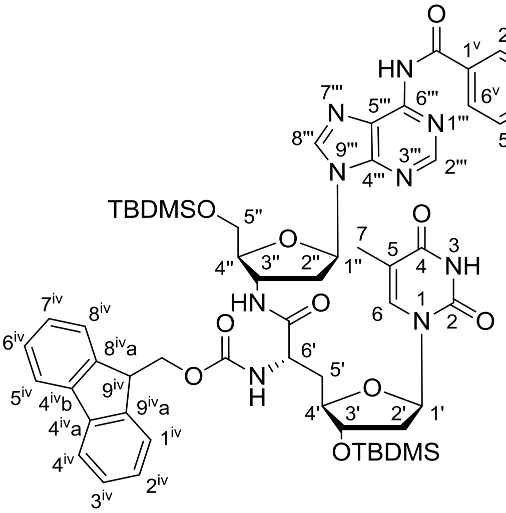

(*S*)-**26**

To a solution of thymidinyl amino acid (*S*)-**9** (690 mg, 1.09 mmol) in CH_2Cl_2 (13 mL), 1-hydroxybenzotriazole (HOBt, 154 mg, 1.14 mmol) and 1-ethyl-3-(3-dimethylaminopropyl)-carbodiimide hydrochloride (EDC, 224 mg, 1.14 mmol) were added. After stirring at room temperature for 15 min, 3'-aminonucleoside **8** (559 mg, 1.20 mmol) was added and stirring at room temperature was continued for 16 h. The solution was diluted with EtOAc (170 mL), washed with sat. NH_4Cl solution (100 mL), water (70 mL) and brine (70 mL) and dried over Na_2SO_4. The solvent was removed under reduced pressure. The resultant crude product was purified by column chromatography (CH_2Cl_2-MeOH 97:3) yielding 835 mg (0.769 mmol,

71%) of (*S*)-**26** as a brownish foam. TLC R_f 0.24 (CH_2Cl_2-MeOH 95:5); mp 158 °C; ^{1}H NMR (500 MHz, C_6D_6, 70 °C): 0.03 (s, 3H, $SiCH_3$), 0.07 (s, 3H, $SiCH_3$), 0.09 (s, 6H, $SiCH_3$), 0.91 (s, 9H, $SiC(CH_3)_3$), 0.94 (s, 9H, $SiC(CH_3)_3$), 1.94 (s, 3H, 7-H), 1.97-2.05 (m, 2H, 2'-H), 2.11-2.20 (m, 1H, 5'-H_a), 2.31-2.40 (m, 1H, 5'-H_b), 2.71 (dd, 2H, *J* = 5.9 Hz, 2''-H), 3.92 (dd, 1H, *J* = 11.2, 3.5 Hz, 5''-H_a), 3.97 (dd, 1H, *J* = 11.2, 2.9 Hz, 5''-H_b), 4.03 (ddd, 1H, *J* = 9.6, 3.4, 3.4 Hz, 4'-H), 4.08 (dd, 1H, *J* = 6.9, 6.9 Hz, 9^{iv}-H), 4.16 (ddd, 1H, *J* = 5.0, 4.4, 4.4 Hz, 3'-H), 4.33 (dd, 1H, *J* = 10.6, 6.8 Hz, FmocCH_2-H_a), 4.34-4.37 (m, 1H, 4''-H), 4.39 (dd, 1H, *J* = 10.6, 7.1 Hz, FmocCH_2-H_b), 4.82 (ddd, 1H, *J* = 7.5, 5.1, 5.1 Hz, 6'-H), 4.86-4.92 (m, 1H, 3''-H), 6.10 (dd, 1H, *J* = 6.4, 6.4 Hz, 1'-H), 6.29-6.37 (m, 1H, 6'-NH), 6.67 (dd, 1H, *J* = 6.4, 6.4 Hz, 1''-H), 7.03 (s, 1H, 6-H), 7.06-7.16 (m, 5H, 2^v-H, 3^v-H, 4^v-H, 5^v-H, 6^v-H), 7.20 (dd, 2H, *J* = 7.4, 7.4 Hz, 3^{iv}-H, 6^{iv}-H), 7.50 (dd, 2H, *J* = 6.7, 6.7 Hz, 2^{iv}-H, 7^{iv}-H), 7.55 (d, 2H, *J* = 7.4 Hz, 4^{iv}-H, 5^{iv}-H), 7.89-8.01 (m, 2H, 1^{iv}-H, 8^{iv}-H), 8.23 (d, 1H, *J* = 5.9 Hz, 3''-NH), 8.52 (s, 1H, 8'''-H), 8.96 (brs, 1H, 2'''-H), 9.42 (brs, 1H, 6'''-NH), 10.78 (brs, 1H, 3-NH); ^{13}C NMR (126 MHz, C_6D_6, 70 °C): -5.6, -5.0, -4.9, 12.0, 17.7, 18.2, 25.6, 25.8, 36.5, 38.2, 39.3, 47.5, 51.3, 52.7, 64.1, 67.0, 75.5, 83.6, 85.0, 86.3, 86.4, 111.0, 119.8, 125.0, 125.0, 126.9, 127.5, 128.1, 128.3, 131.7, 134.3, 141.4, 141.4, 144.1, 144.2, 150.0, 150.7, 151.6, 152.5, 156.0, 164.0, 171.0; IR (ATR) ν 1685, 1609, 1447, 1250, 1223, 1075, 1037, 831, 779; UV (MeCN) λ_{max} (log ε) 203 (4.55), 266 (4.19); $[\alpha]_D^{20}$ -18.1 (c 1.0, $CHCl_3$); MS (ESI^+) *m/z* calcd for $C_{56}H_{72}N_9O_{10}Si_2$ 1086.4935 ($M+H^+$), found 1086.4940.

Bis-*O*-TBDMS-protected (6'*R*)-thymidinyl amino acid (3'-amino-2',3'-dideoxy-3'-yl)-amide (*R*)-26

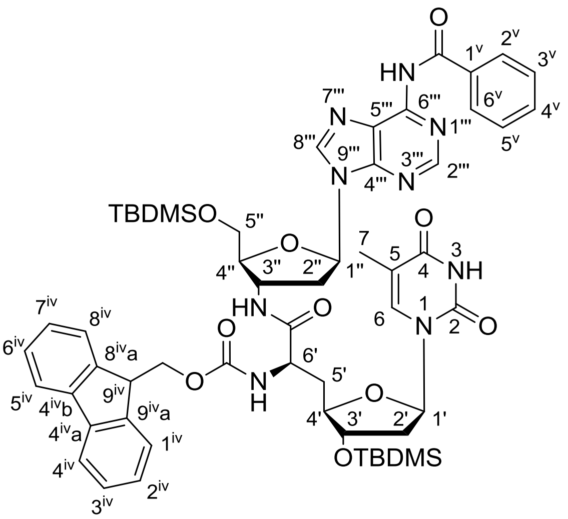

(*R*)-**26**

The synthesis of (*R*)-**26** was performed according to the protocol for the synthesis of (*S*)-**26** with didehydro thymidinyl amino acid (*R*)-**9** (530 mg, 0.834 mmol), 3'-aminonucleoside **8** (429 mg, 0.918 mmol), EDC (172 mg, 0.878 mmol), HOBt (118 mg, 0.878 mmol) and CH_2Cl_2 (10 mL) to yield 620 mg (0.571 mmol, 68%) of (*R*)-**26** as a brownish foam. TLC R_f 0.24 (CH_2Cl_2-MeOH 95:5); mp 157 °C; ^{1}H NMR (500 MHz, C_6D_6, 70 °C): 0.05 (s, 3H, $SiCH_3$), 0.07 (s, 9H, $SiCH_3$), 0.91 (s, 9H, $SiC(CH_3)_3$), 0.92 (s, 9H, $SiC(CH_3)_3$), 1.81 (s, 3H, 7-H), 1.88 (ddd, 1H, J = 13.3, 6.3, 2.6 Hz, 2'-H_a), 1.97-2.04 (m, 1H, 5'-H_a), 2.32 (ddd, 1H, J = 13.8, 11.2, 2.9 Hz, 5'-H_b), 2.57 (ddd, 1H, J = 13.3, 7.1, 6.3 Hz, 2'-H_b), 2.72-2.79 (m, 2H, 2''-H), 3.87 (d, 2H, J = 2.9 Hz, 5''-H), 4.09-4.17 (m, 2H, 3'-H, 9^{iv}-H), 4.19-4.26 (m, 2H, 4'-H, 4''-H), 4.37 (d, 2H, J = 7.1 Hz, $FmocCH_2$), 4.85-4.94 (m, 2H, 6'-H, 3''-H), 5.67 (dd, 1H, J = 7.1, 7.1 Hz, 1'-H), 6.19 (d, 1H, J = 8.4 Hz, 6'-NH), 6.56 (s, 1H, 6-H), 6.74 (dd, 1H, J = 6.6, 6.6 Hz, 1''-H), 7.08-7.24 (m, 7H, 3^{iv}-H, 6^{iv}-H, 2^{v}-H, 3^{v}-H, 4^{v}-H, 5^{v}-H, 6^{v}-H), 7.52 (dd, 2H, J = 9.7, 7.8 Hz, 2^{iv}-H, 7^{iv}-H), 7.56 (d, 2H, J = 7.6 Hz, 4^{iv}-H, 5^{iv}-H), 7.88 (d, 1H, J = 7.1 Hz, 3''-NH), 7.93-8.03 (m, 2H, 1^{iv}-H, 8^{iv}-H), 8.51 (s, 1H, 8'''-H), 9.12 (brs, 1H, 2'''-H), 9.49 (brs, 1H, 6'''-NH), 10.95 (brs, 1H, 3-NH); ^{13}C NMR (126 MHz, C_6D_6, 50 °C): -5.7, -5.0,

11.9, 17.8, 18.2, 25.6, 25.8, 37.8, 38.1, 38.6, 47.5, 51.2, 52.4, 64.2, 67.1, 76.2, 84.2, 84.3, 86.8, 90.7, 110.6, 119.8, 123.8, 125.1, 126.9, 127.7, 127.9, 128.3, 131.7, 136.9, 138.7, 141.0, 141.4, 144.2, 144.3, 150.2, 150.6, 151.5, 152.8, 156.5, 164.0, 172.1; IR (ATR) ν 1680, 1609, 1447, 1250, 1223, 1070, 1045, 832, 774; UV (MeCN) λ_{max} (log ε) 203 (5.05), 266 (4.68); $[\alpha]_D^{20}$ -111.2 (c 1.1, $CHCl_3$); MS (ESI^+) *m/z* calcd for $C_{56}H_{72}N_9O_{10}Si_2$ 1086.4935 ($M+H^+$), found 1086.4935.

Bis-*O*-deprotected (6'*S*)-thymidinyl amino acid (3'-amino-2',3'-dideoxy-3'-yl)-amide (*S*)-27

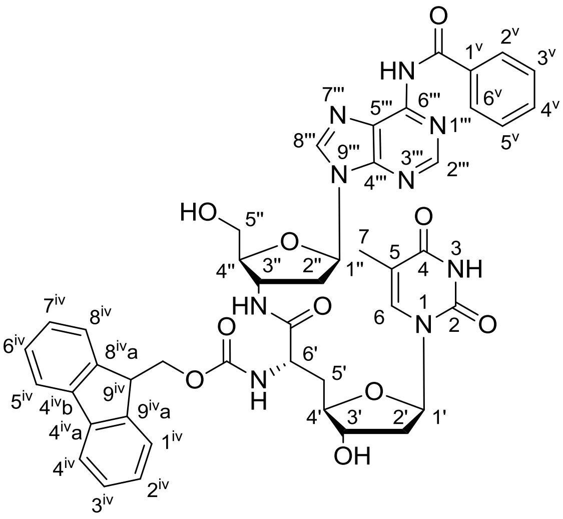

(*S*)-**27**

To a solution of bis-*O*-TBDMS-protected dimer (*S*)-**26** (650 mg, 0.598 mmol) in MeOH (10 mL), ammonium fluoride (249 mg, 6.73 mmol) was added and the reaction mixture was stirred for 4.5 h at 60 °C. To this solution, silica (*ca.* one third of the volume) was added and the solvent was removed under reduced pressure. The resultant crude product (adsorbed on silica) was purified by column chromatography (CH_2Cl_2-MeOH 9:1) yielding 340 mg (0.396 mmol, 66%) of (*S*)-**27** as a yellowish solid. TLC R_f 0.16 (CH_2Cl_2-MeOH 9:1); mp decomposition > 155 °C; ^1H NMR (500 MHz, pyridine-d_5, 50 °C): 2.01 (s, 3H, 7-H), 2.32-2.46 (m, 2H, 2'-H_a, 5'-H_a), 2.56 (ddd, 1H, *J* = 13.4, 6.5, 3.4 Hz, 2'-H_b), 2.73 (ddd, 1H,

J = 13.0, 9.4, 3.5 Hz, 5'-H_b), 2.82 (ddd, 1H, *J* = 13.4, 6.7, 6.7 Hz, 2"-H_a), 3.09 (ddd, 1H, *J* = 13.4, 7.9, 5.4 Hz, 2"-H_b), 4.17 (dd, 1H, *J* = 12.1, 3.6 Hz, 5"-H_a), 4.22 (dd, 1H, *J* = 12.1, 3.0 Hz, 5"-H_b), 4.31 (dd, 1H, *J* = 6.6, 6.6 Hz, 9^{iv}-H), 4.49-4.58 (m, 5H, 3'-H, 4'-H, 4"-H, FmocCH_2), 5.07 (ddd, 1H, *J* = 8.5, 5.8, 5.8 Hz, 6'-H), 5.33 (dddd, 1H, *J* = 6.7, 6.7, 6.7, 6.7 Hz, 3"-H), 6.76 (dd, 1H, *J* = 6.7, 5.4 Hz, 1"-H), 6.87 (dd, 1H, *J* = 6.5, 6.5 Hz, 1'-H), 7.25-7.30 (m, 2H, 2^{iv}-H, 7^{iv}-H), 7.38 (ddd, 2H, *J* = 7.5, 7.5, 3.9 Hz, 3^{iv}-H, 6^{iv}-H), 7.43 (dd, 2H, *J* = 7.3, 7.3 Hz, 3^{v}-H, 5^{v}-H), 7.49 (dd, 1H, *J* = 7.3, 7.3 Hz, 4^{v}-H), 7.54 (s, 1H, 6-H), 7.68 (d, 2H, *J* = 7.2 Hz, 1^{iv}-H, 8^{iv}-H), 7.82 (dd, 2H, *J* = 7.6, 2.4 Hz, 4^{iv}-H, 5^{iv}-H), 8.28 (d, 2H, *J* = 7.3 Hz, 2^{v}-H, 6^{v}-H), 8.84-8.91 (m, 1H, 6'-NH), 8.86 (s, 1H, 8'"-H), 8.98 (s, 1H, 2'"-H), 9.73 (d, 1H, *J* = 5.5 Hz, 3"-NH), 11.86 (brs, 1H, 3-NH); ^{13}C NMR (126 MHz, pyridine-d_5, 50 °C): 12.1, 37.4, 38.3, 39.6, 47.7, 50.7, 53.3, 62.4, 66.6, 74.6, 84.3, 84.8, 85.2, 86.7, 111.0, 120.2, 120.2, 122.8, 125.3, 125.4, 127.2, 127.8, 128.5, 128.8, 132.1, 135.5, 135.7, 141.5, 142.3, 144.4, 144.4, 151.2, 151.5, 151.9, 152.2, 156.9, 164.6, 172.4; IR (ATR) ν 1681, 1666, 1609, 1513, 1447, 1250, 1222, 1050, 703; UV (MeCN) λ_{max} (log ε) 199 (3.94), 256 (3.56); $[\alpha]_D^{20}$ +6.7 (c 1.1, pyridine); MS (ESI$^+$) *m/z* calcd for $C_{44}H_{44}N_9O_{10}$ 858.3206 (M+H$^+$), found 858.3207.

Bis-*O*-deprotected (6'*R*)-thymidinyl amino acid (3'-amino-2',3'-dideoxy-3'-yl)-amide (*R*)-27

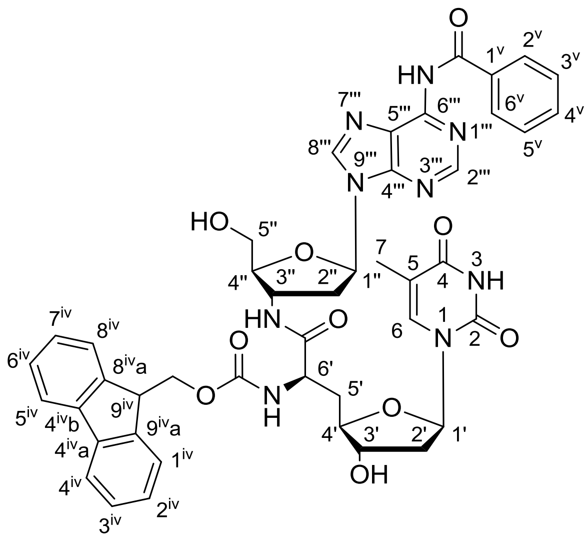

(*R*)-**27**

The synthesis of (*R*)-**27** was performed according to the protocol for the synthesis of (*S*)-**27** with bis-*O*-TBDMS-protected dimer (*R*)-**26** (550 mg, 0.506 mmol), ammonium fluoride (198 mg, 5.35 mmol) and MeOH (10 mL) to yield 287 mg (0.335 mmol, 66%) of (*R*)-**27** as a yellowish solid. TLC R_f 0.16 (CH_2Cl_2-MeOH 9:1); mp decomposition > 145 °C; ^{1}H NMR (500 MHz, pyridine-d_5, 50 °C): 2.01 (s, 3H, 7-H), 2.56-2.64 (m, 3H, 2'-H, 5'-H_a), 2.69 (ddd, 1H, *J* = 14.6, 9.3, 5.4 Hz, 5'-H_b), 2.89 (ddd, 1H, *J* = 13.4, 6.6, 6.4 Hz, 2"-H_a), 3.14 (ddd, 1H, *J* = 13.4, 7.7, 5.7 Hz, 2"-H_b), 4.13 (dd, 1H, *J* = 12.1, 3.5 Hz, 5"-H_a), 4.19 (dd, 1H, *J* = 12.1, 2.8 Hz, 5"-H_b), 4.29 (dd, 1H, *J* = 6.7, 6.7 Hz, 9^{iv}-H), 4.49-4.58 (m, 5H, 3'-H, 4'-H, 4"-H, $FmocCH_2$), 5.10 (ddd, 1H, *J* = 8.0, 5.4, 5.4 Hz, 6'-H), 5.30 (dddd, 1H, *J* = 6.6, 6.6, 6.6, 6.6 Hz, 3"-H), 6.70 (dd, 1H, *J* = 6.6, 6.6 Hz, 1'-H), 6.81 (dd, 1H, *J* = 6.4, 6.4 Hz, 1"-H), 7.29 (dd, 2H, *J* = 7.2, 7.2 Hz, 2^{iv}-H, 7^{iv}-H), 7.39 (dd, 2H, *J* = 7.5, 7.5 Hz, 3^{iv}-H, 6^{iv}-H), 7.43 (dd, 2H, *J* = 7.3, 7.3 Hz, 3^{v}-H, 5^{v}-H), 7.49 (dd, 1H, *J* = 7.4, 7.4 Hz, 4^{v}-H), 7.55 (s, 1H, 6-H), 7.67 (d, 1H, *J* = 7.2 Hz, 1H, 1^{iv}-H), 7.69 (d, 1H, *J* = 7.2 Hz, 8^{iv}-H), 7.82 (d, 2H, *J* = 7.6 Hz, 4^{iv}-H, 5^{iv}-H), 8.28 (d, 2H, *J* = 7.3 Hz, 2^{v}-H, 6^{v}-H), 8.84-8.91 (m, 1H, 6'-NH), 8.92 (s, 1H, 8'''-H), 9.00 (s, 1H, 2'''-H), 9.33 (d, *J* = 6.6 Hz, 1H, 3"-NH), 11.85 (brs, 1H, 3-NH); ^{13}C NMR

(126 MHz, pyridine-d_5, 50 °C): 12.3, 37.1, 38.4, 39.8, 47.6, 50.9, 53.7, 62.4, 66.7, 74.5, 83.8, 84.8, 86.0, 86.7, 110.8, 120.1, 122.8, 125.4, 125.7, 127.3, 127.8, 128.5, 128.8, 132.1, 135.5, 136.4, 141.5, 142.2, 144.3, 144.4, 151.3, 151.4, 152.0, 152.2, 157.0, 164.5, 173.2; IR (ATR) ν 1685, 1666, 1609, 1513, 1452, 1442, 1250, 1223, 1065, 703; UV (MeCN) λ_{max} (log ε) 198 (4.22), 256 (3.86); $[\alpha]_D^{20}$ +27.6 (c 1.0, pyridine); MS (ESI^+) *m/z* calcd for $C_{44}H_{43}N_9NaO_{10}$ 880.3025 ($M+Na^+$), found 880.3022.

O-DMTr-protected (6'S)-thymidinyl amino acid (3'-amino-2',3'-dideoxy-3'-yl)-amide (S)-28

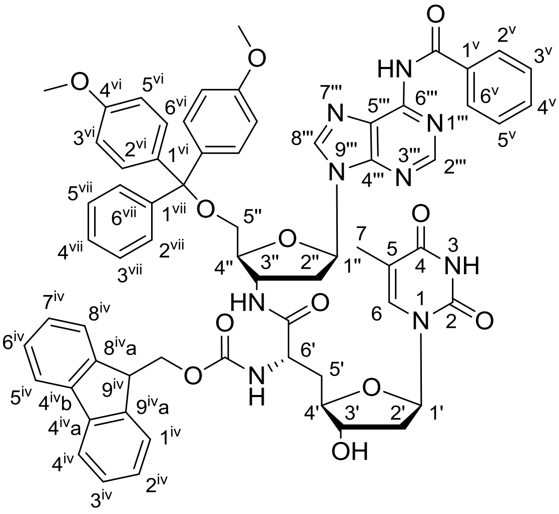

(*S*)-**28**

Bis-*O*-deprotected dimer (*S*)-**27** (320 mg, 0.373 mmol) was coevaporated with pyridine (3 x 2 mL) and then dissolved in pyridine (1 mL). To this solution, DMAP (4.5 mg, 37 µmol) and 4,4'-dimethoxytrityl chloride (DMTrCl, 138 mg, 0.410 mmol) were added and the resultant solution was stirred at room temperature for 16 h. The solvent was removed under reduced pressure and the residue was dissolved in EtOAc (30 mL). This solution was washed with sat. $NaHCO_3$ solution (20 mL), water (20 mL) and brine (20 mL) and dried over Na_2SO_4. The solvent was removed under reduced pressure. The resultant crude product was purified by column chromatography (CH_2Cl_2-MeOH-pyridine 92:7:1) yielding 310 mg (0.267 mmol,

72%) of (*S*)-**28** as a colorless foam. TLC R_f 0.27 (CH_2Cl_2-MeOH 98:2); mp 130 °C; ^{1}H NMR (500 MHz, pyridine-d_5, 50 °C): 2.00 (s, 3H, 7-H), 2.30-2.43 (m, 2H, 2'-H_a, 5'-H_a), 2.55 (ddd, 1H, *J* = 13.4, 6.6, 3.6 Hz, 2'-H_b), 2.70 (ddd, 1H, *J* = 13.6, 9.0, 4.4 Hz, 5'-H_b), 2.85 (ddd, 1H, *J* = 13.4, 6.8, 6.7 Hz, 2''-H_a), 3.21 (ddd, 1H, *J* = 13.4, 8.0, 5.6 Hz, 2''-H_b), 3.67 (s, 3H, OCH_3), 3.68 (s, 3H, OCH_3), 3.73-3.81 (m, 2H, 5''-H), 4.30 (dd, 1H, *J* = 6.8, 6.8 Hz, 9^{iv}-H), 4.47 (ddd, 1H, *J* = 9.5, 3.8 Hz, 4'-H), 4.53 (d, 1H, *J* = 6.8 Hz, FmocCH_2-H_a), 4.55 (d, 1H, *J* = 6.8 Hz, FmocCH_2-H_b), 4.67-4.72 (m, 1H, 4''-H), 5.04 (ddd, 1H, *J* = 8.3, 8.3, 6.3 Hz, 6'-H), 5.31 (dddd, 1H, *J* = 6.8, 6.8, 6.8, 6.8 Hz, 3''-H), 6.75 (dd, 1H, *J* = 6.7, 5.6 Hz, 1''-H), 6.82 (dd, 1H, *J* = 6.6, 6.6 Hz, 1'-H), 6.93 (dd, 4H, *J* = 9.0, 2.5 Hz, 3^{vi}-H, 5^{vi}-H), 7.18-7.23 (m, 2H, 2^{v}-H, 6^{v}-H), 7.26 (ddd, 1H, *J* = 7.5, 7.5, 0.8 Hz, 2^{iv}-H), 7.27 (ddd, 1H, *J* = 7.5, 7.5, 1.1 Hz, 7^{iv}-H), 7.34 (dd, 2H, *J* = 7.8, 7.8 Hz, 2^{vii}-H, 6^{vii}-H), 7.38 (ddd, 2H, *J* = 7.5, 7.5, 7.5 Hz, 3^{iv}-H, 6^{iv}-H), 7.42 (dd, 2H, *J* = 7.2, 7.2 Hz, 3^{v}-H, 5^{v}-H), 7.49 (dd, 1H, *J* = 7.4, 7.4 Hz, 4^{v}-H), 7.54-7.58 (m, 5H, 6-H, 2^{vi}-H, 6^{vi}-H), 7.65-7.69 (m, 2H, 1^{iv}-H, 8^{iv}-H), 7.69-7.73 (m, 2H, 3^{vii}-H, 5^{vii}-H), 7.82 (dd, 2H, *J* = 7.6, 2.9 Hz, 4^{iv}-H, 5^{iv}-H), 8.71 (s, 1H, 8'''-H), 8.84 (s, 1H, 2'''-H), 8.85-8.92 (m, 1H, 6'-NH), 9.63 (brs, 1H, 3''-NH), 12.94 (brs, 1H, 3-NH); ^{13}C NMR (126 MHz, pyridine-d_5, 50 °C): 12.2, 37.3, 37.6, 39.7, 47.7, 51.3, 53.3, 55.0, 64.5, 66.6, 74.5, 84.4, 84.8, 85.1, 86.7, 111.0, 113.5, 120.2, 123.8, 125.4, 125.4, 126.9, 127.2, 127.8, 128.0, 128.5, 128.6, 128.8, 130.4, 130.4, 132.1, 135.5, 135.7, 136.4, 141.5, 142.2, 144.4, 144.4, 145.5, 150.0, 151.3, 151.5, 152.2, 158.9, 164.6, 172.2; IR (ATR) ν 1680, 1509, 1447, 1247, 1223, 1174, 1027, 822, 703; UV (MeCN) λ_{max} (log ε) 197 (4.76), 255 (4.23); $[\alpha]_D^{20}$ -9.9 (c 1.0, $CHCl_3$); MS (ESI^+) *m/z* calcd for $C_{65}H_{62}N_9O_{12}$ 1160.4512 ($M+H^+$), found 1160.4509.

***O*-DMTr-protected (6'*R*)-thymidinyl amino acid (3'-amino-2',3'-dideoxy-3'-yl)-amide (*R*)-28**

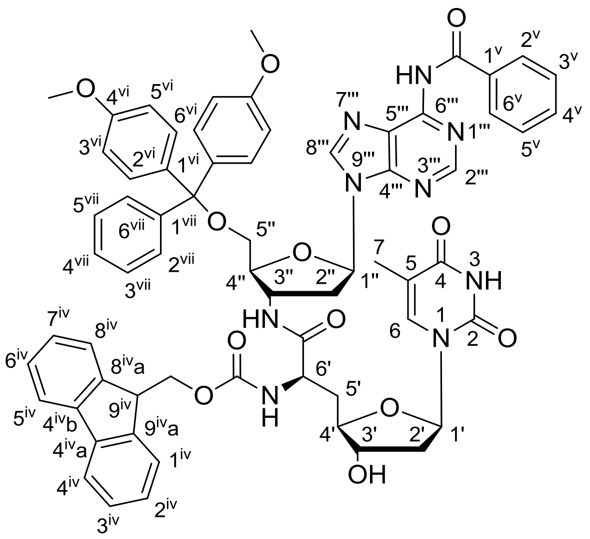

(*R*)-**28**

The synthesis of (*R*)-**28** was performed according to the protocol for the synthesis of (*S*)-**28** with bis-*O*-deprotected dimer (*R*)-**27** (270 mg, 0.315 mmol), 4,4'-dimethoxytrityl chloride (DMTrCl, 117 mg, 0.347 mmol), DMAP (3.0 mg, 25 µmol) and pyridine (0.7 mL) to yield 295 mg (0.254 mmol, 81%) of (*R*)-**28** as a colorless foam. TLC R_f 0.27 (CH_2Cl_2-MeOH 98:2); mp 125 °C; ^1H NMR (500 MHz, pyridine-d_5, 50 °C): 1.98 (s, 3H, 7-H), 2.53-2.61 (m, 3H, 2'-H, 5'-H_a), 2.67 (ddd, 1H, J = 14.6, 9.5, 5.4 Hz, 5'-H_b), 2.91 (ddd, 1H, J = 13.4, 6.5, 6.3 Hz, 2''-H_a), 3.25 (ddd, 1H, J = 13.4, 7.8, 5.4 Hz, 2''-H_b), 3.64 (s, 3H, OCH_3), 3.66 (s, 3H, OCH_3), 3.71 (dd, 1H, J = 10.4, 3.5 Hz, 5''-H_a), 3.75 (dd, 1H, J = 10.4, 5.2 Hz, 5''-H_b), 4.29 (dd, 1H, J = 7.0, 7.0 Hz, 9^{iv}-H), 4.47-4.57 (m, 4H, 3'-H, 4'-H, FmocCH_2), 4.67-4.72 (m, 1H, 4''-H), 5.08 (ddd, 1H, J = 8.0, 8.0, 5.9 Hz, 6'-H), 5.32 (dddd, 1H, J = 5.4, 5.4, 5.4, 5.4 Hz, 3''-H), 6.69 (dd, 1H, J = 6.5, 6.5 Hz, 1'-H), 6.78 (dd, 1H, J = 6.3, 6.3 Hz, 1''-H), 6.90 (d, 4H, J = 8.9 Hz, 3^{vi}-H, 5^{vi}-H), 7.17-7.21 (m, 2H, 2^{v}-H, 6^{v}-H), 7.25-7.33 (m, 4H, 2^{iv}-H, 7^{iv}-H, 2^{vii}-H, 6^{vii}-H), 7.36-7.44 (m, 4H, 3^{iv}-H, 6^{iv}-H, 3^{v}-H, 5^{v}-H), 7.49 (dd, 1H, J = 7.4, 7.4 Hz, 4^{v}-H), 7.51-7.58 (m, 5H, 6-H, 2^{vi}-H, 6^{vi}-H), 7.63-7.70 (m, 4H, 1^{iv}-H, 8^{iv}-H, 3^{vii}-H, 5^{vii}-H), 7.82 (d, 2H, J = 7.6 Hz, 4^{iv}-H, 5^{iv}-H), 8.25-8.29 (m, 2H, 1^{iv}-H, 8^{iv}-H), 8.67-8.72 (m, 2H, 6'-NH, 8'''-H),

8.90 (m, 1H, 2'''-H), 9.27-9.32 (m, 1H, 3''-NH), 11.84 (brs, 1H, 6'''-NH), 12.86 (brs, 1H, 3-NH); ^{13}C NMR (126 MHz, pyridine-d$_5$, 50 °C): 12.3, 37.3, 37.9, 39.8, 47.6, 51.3, 53.7, 55.0, 64.5, 66.7, 74.5, 83.8, 84.4, 84.8, 86.0, 86.7, 110.7, 113.5, 120.2, 123.8, 125.4, 125.8, 126.9, 127.3, 127.8, 128.0, 128.5, 128.6, 128.8, 130.4, 132.1, 135.5, 135.7, 136.3, 141.5, 142.1, 144.3, 144.5, 145.4, 150.0, 151.3, 151.4, 152.2, 158.9, 164.5, 172.9; IR (ATR) ν 1678, 1509, 1437, 1247, 1217, 1174, 1070, 1032, 703; UV (MeCN) λ_{max} (log ε) 199 (4.61), 264 (4.01); $[\alpha]_D^{20}$ -27.4 (c 1.0, CHCl$_3$); MS (ESI$^+$) *m/z* calcd for $C_{65}H_{62}N_9O_{12}$ 1160.4512 (M+H$^+$), found 1160.4509.

^{1}H, ^{13}C and ^{31}P NMR spectra of synthesized compounds

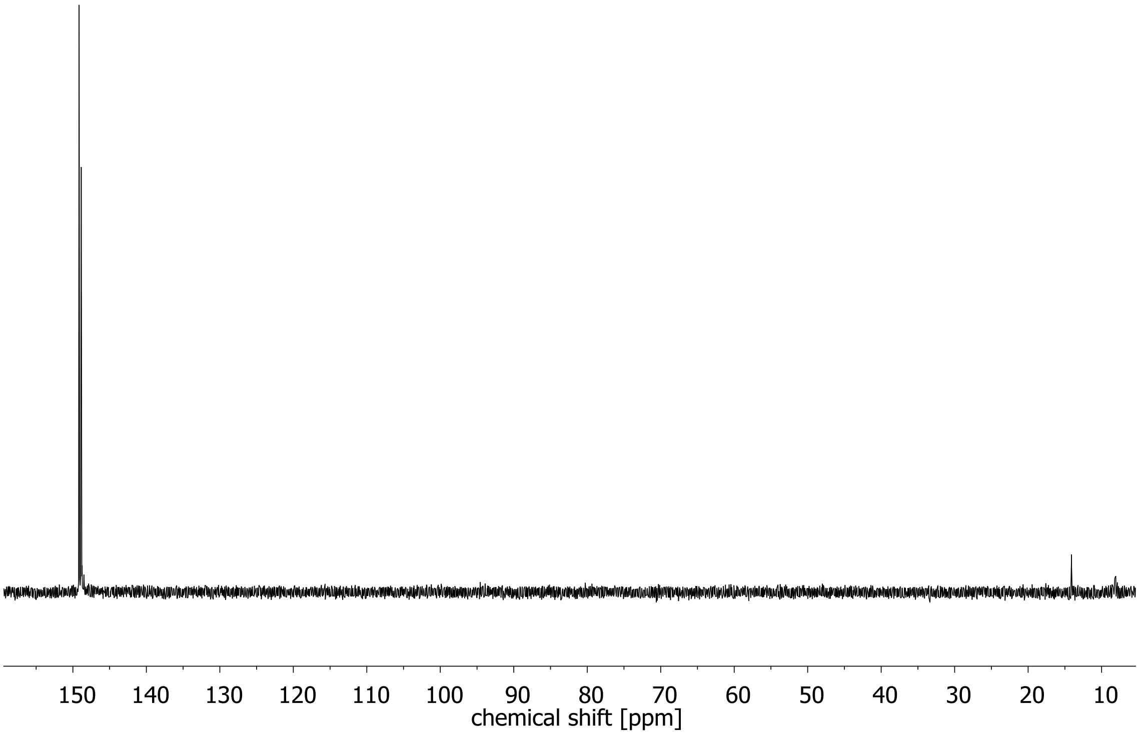

^{31}P NMR spectrum of (*S*)-**7** (121 MHz, pyridine-d_5)

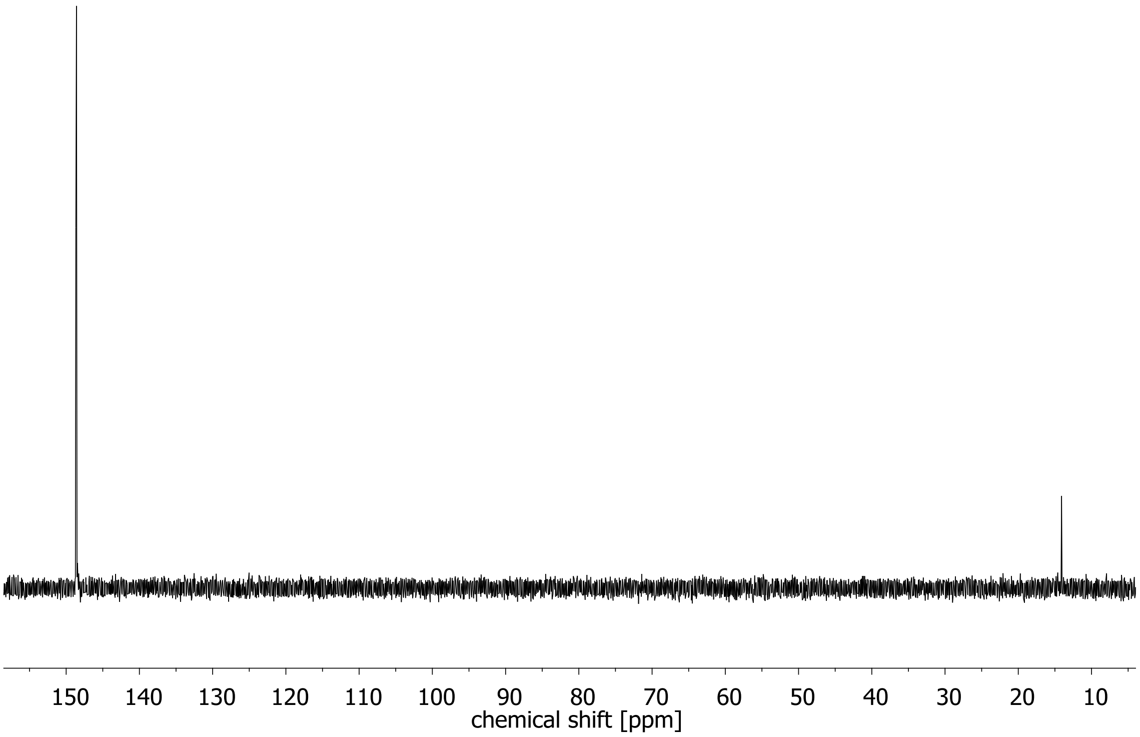

^{31}P NMR spectrum of (*R*)-**7** (121 MHz, pyridine-d_5)

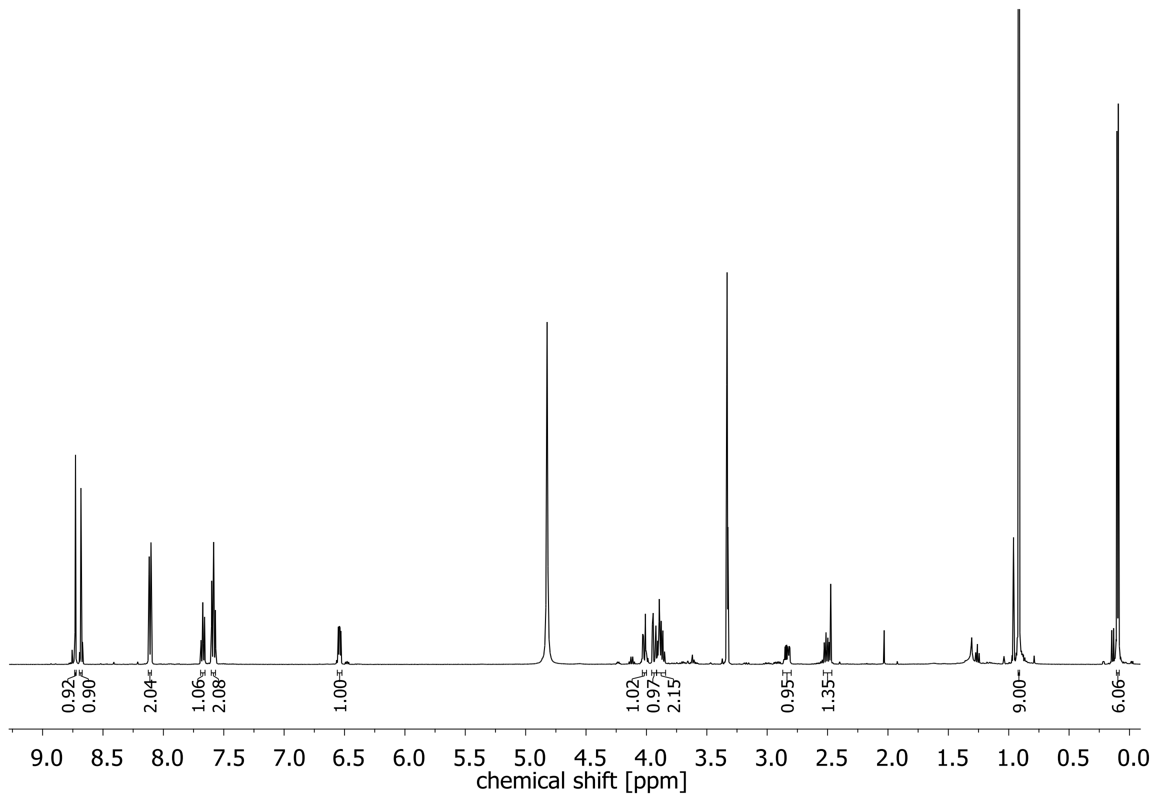

^{1}H NMR spectrum of **8** (300 MHz, $CDCl_3$)

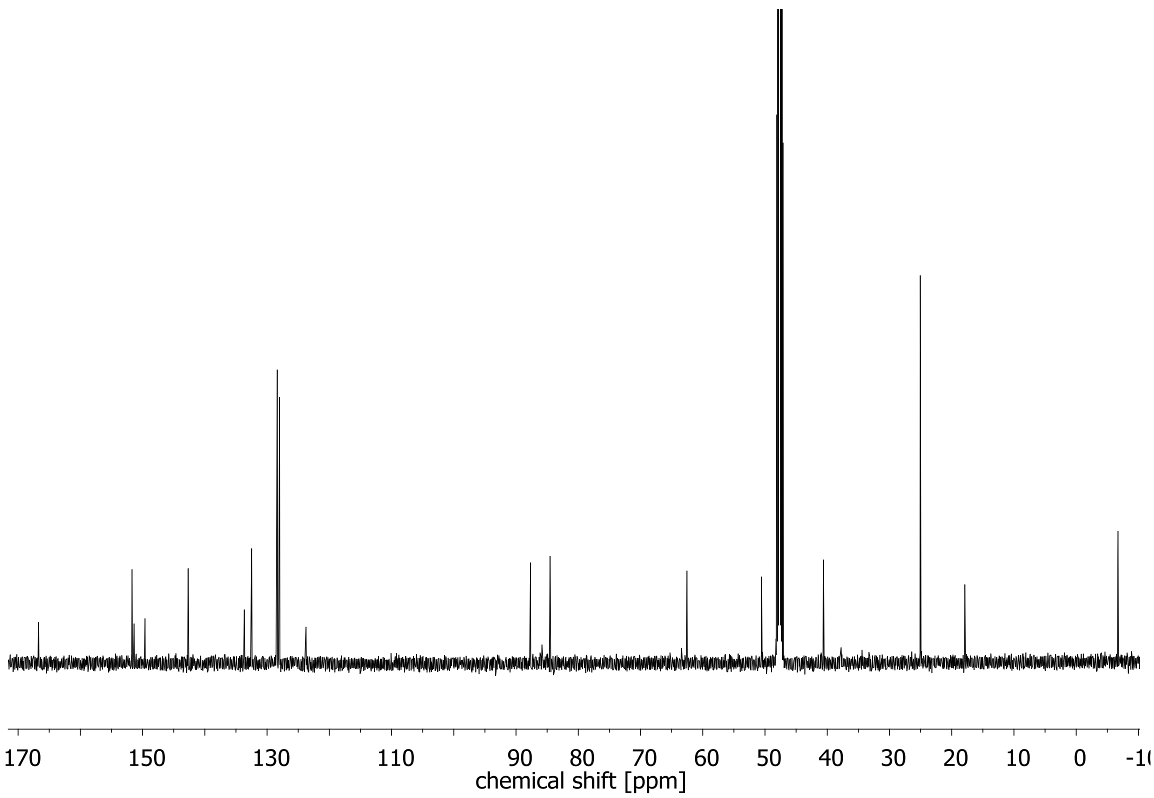

^{13}C NMR spectrum of **8** (75 MHz, $CDCl_3$)

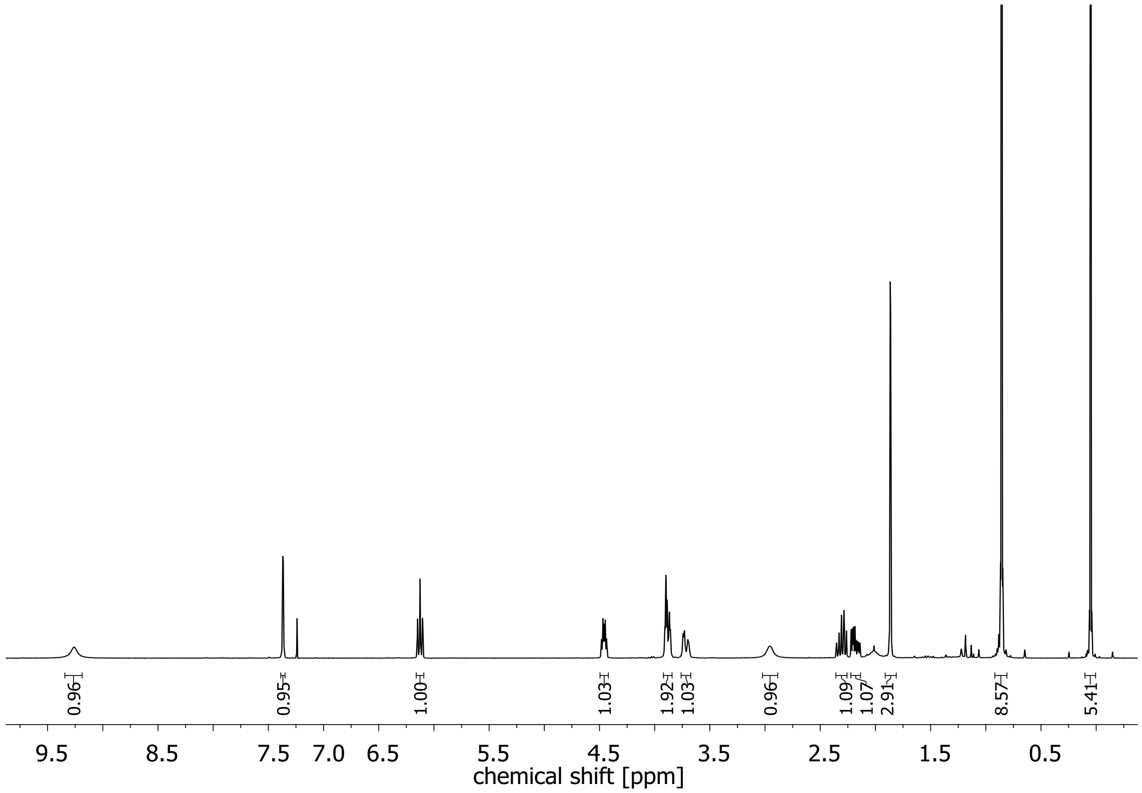

^{1}H NMR spectrum of **16** (300 MHz, $CDCl_3$)

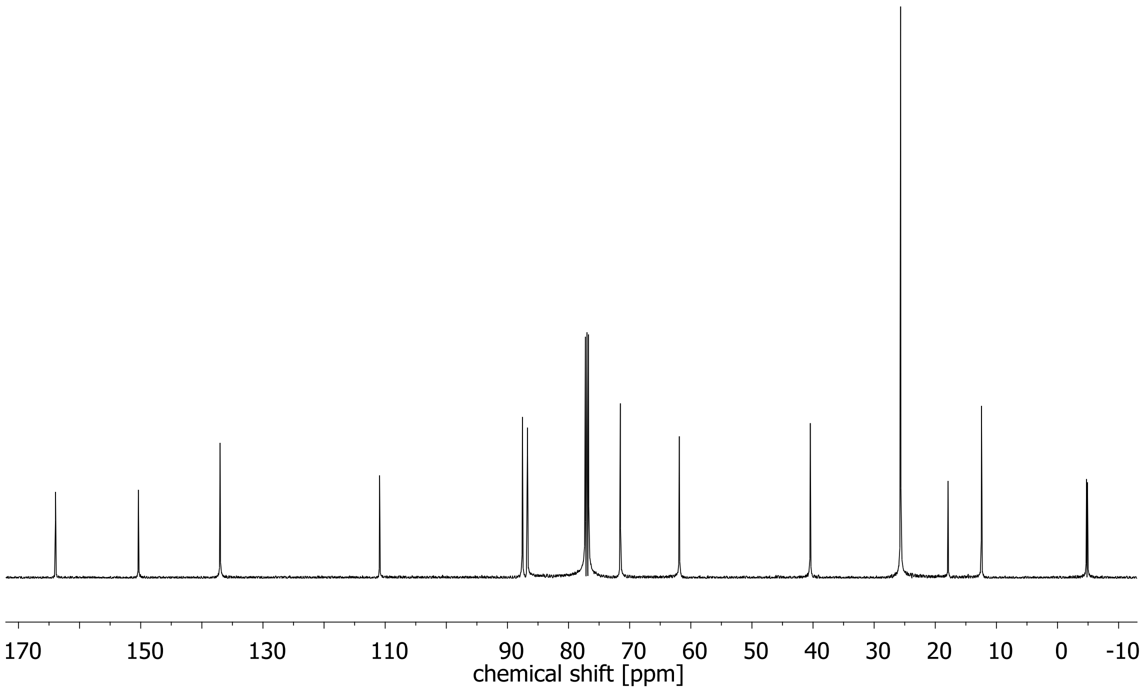

^{13}C NMR spectrum of **16** (75 MHz, $CDCl_3$)

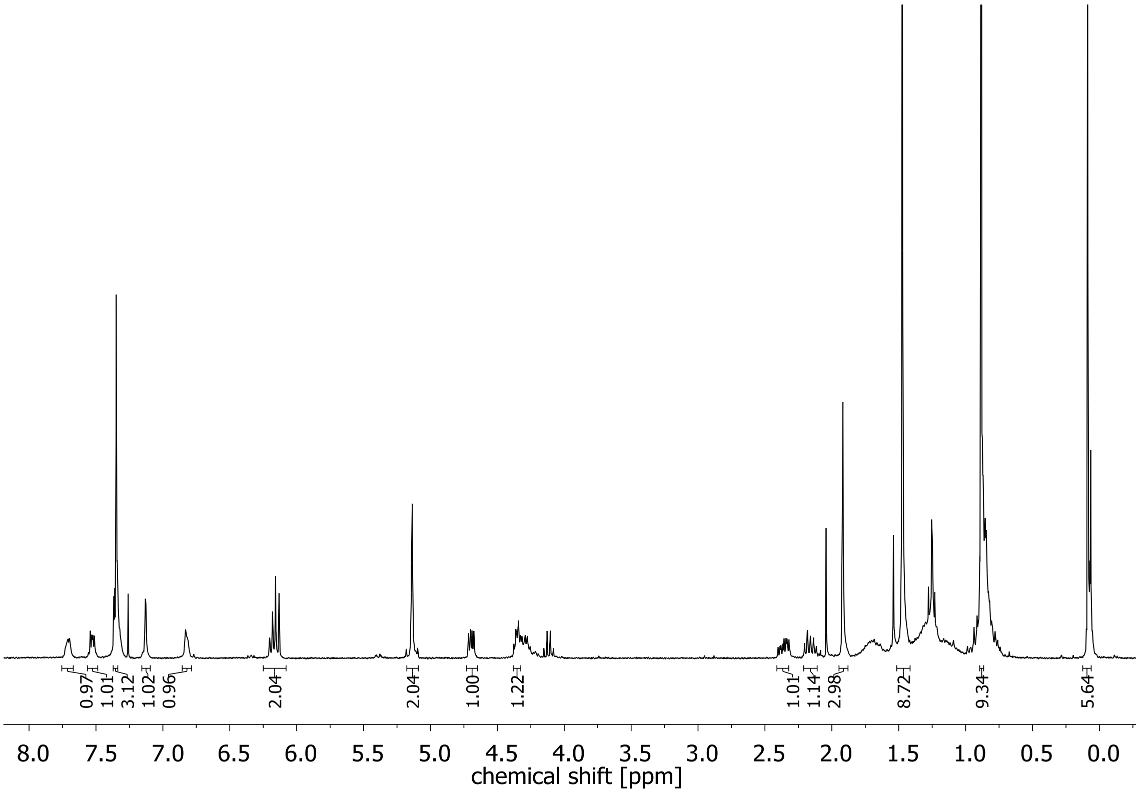

^{1}H NMR spectrum of *Z*-**18** (300 MHz, $CDCl_3$)

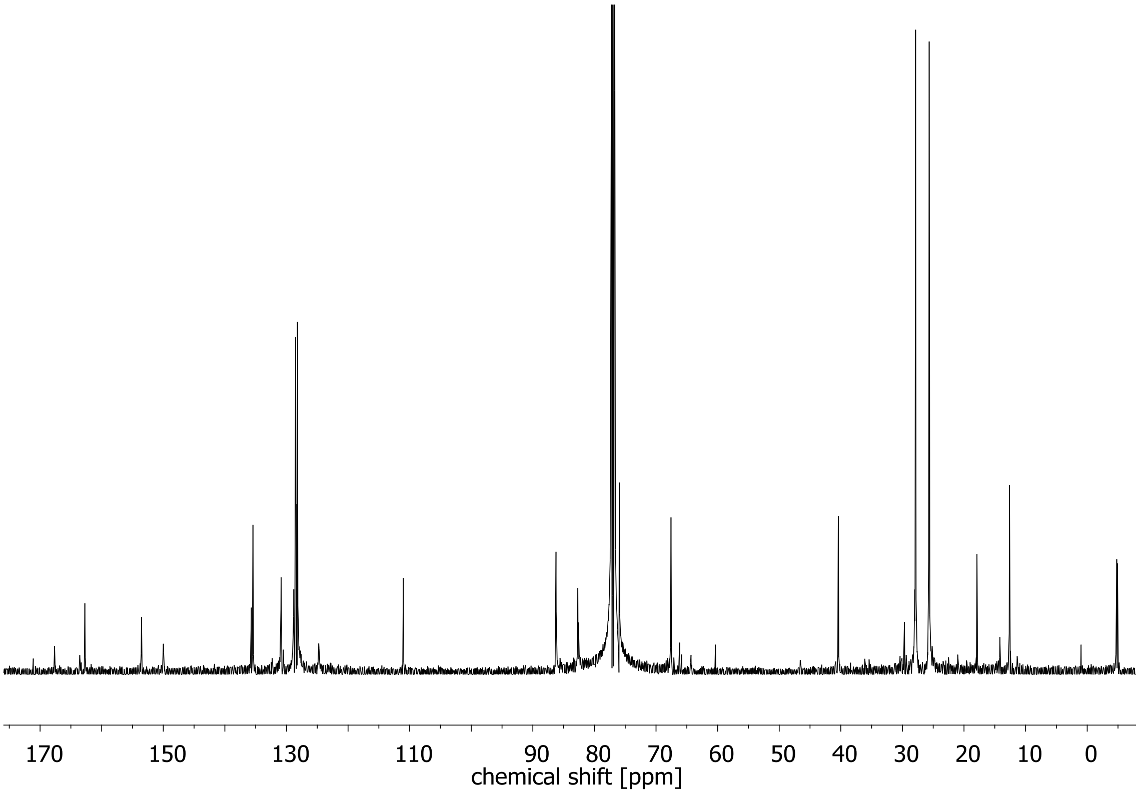

^{13}C NMR spectrum of *Z*-**18** (75 MHz, $CDCl_3$)

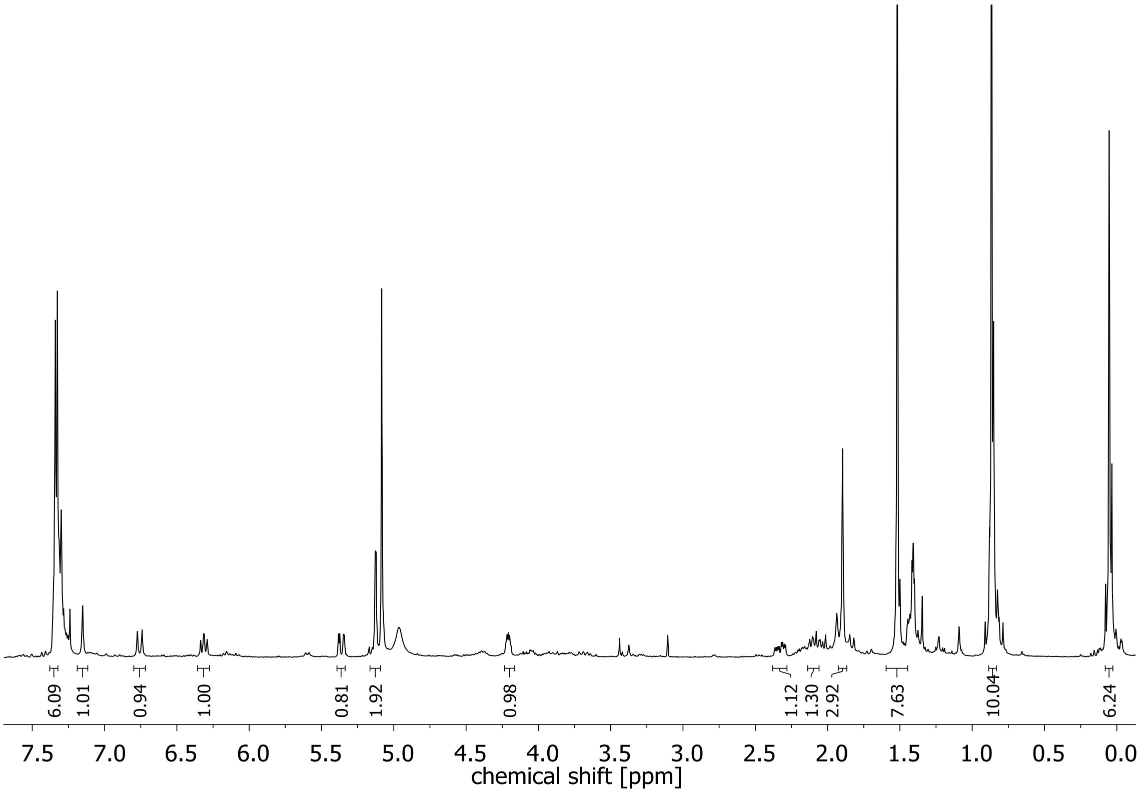

^{1}H NMR spectrum of *E*-**18** (300 MHz, $CDCl_3$)

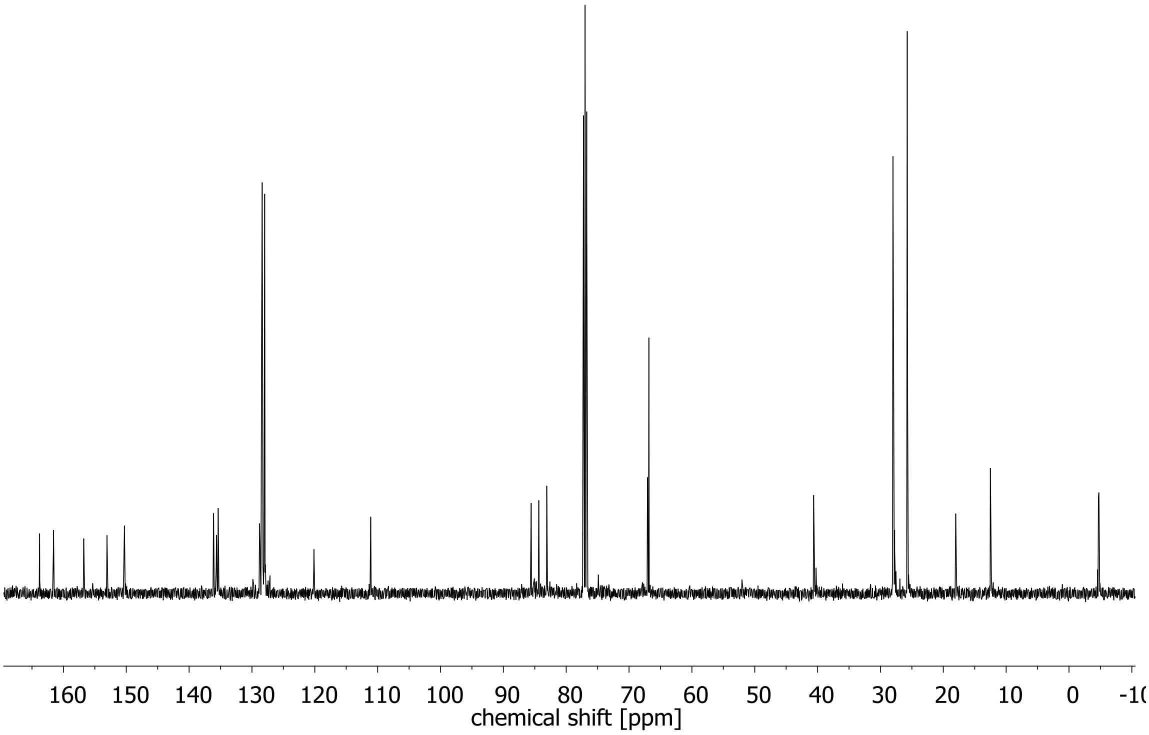

^{13}C NMR spectrum of *E*-**18** (75 MHz, $CDCl_3$)

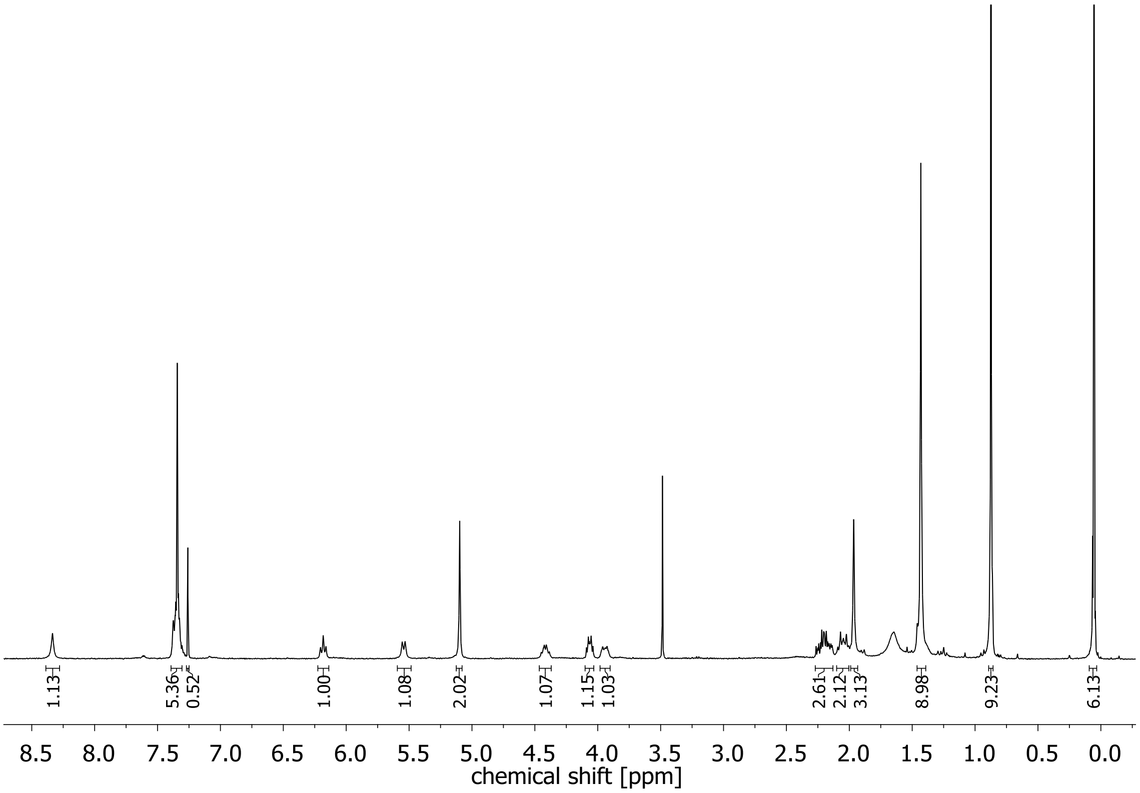

^{1}H NMR spectrum of (*S*)-**20** (300 MHz, $CDCl_3$)

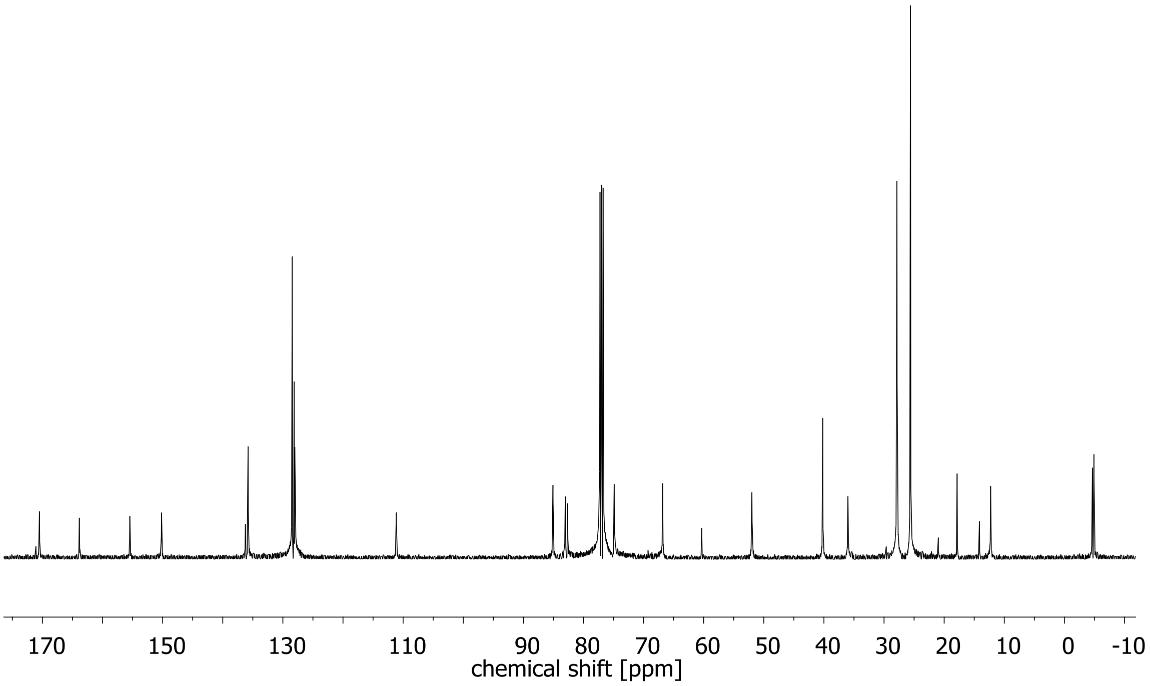

^{13}C NMR spectrum of (*S*)-**20** (75 MHz, $CDCl_3$)

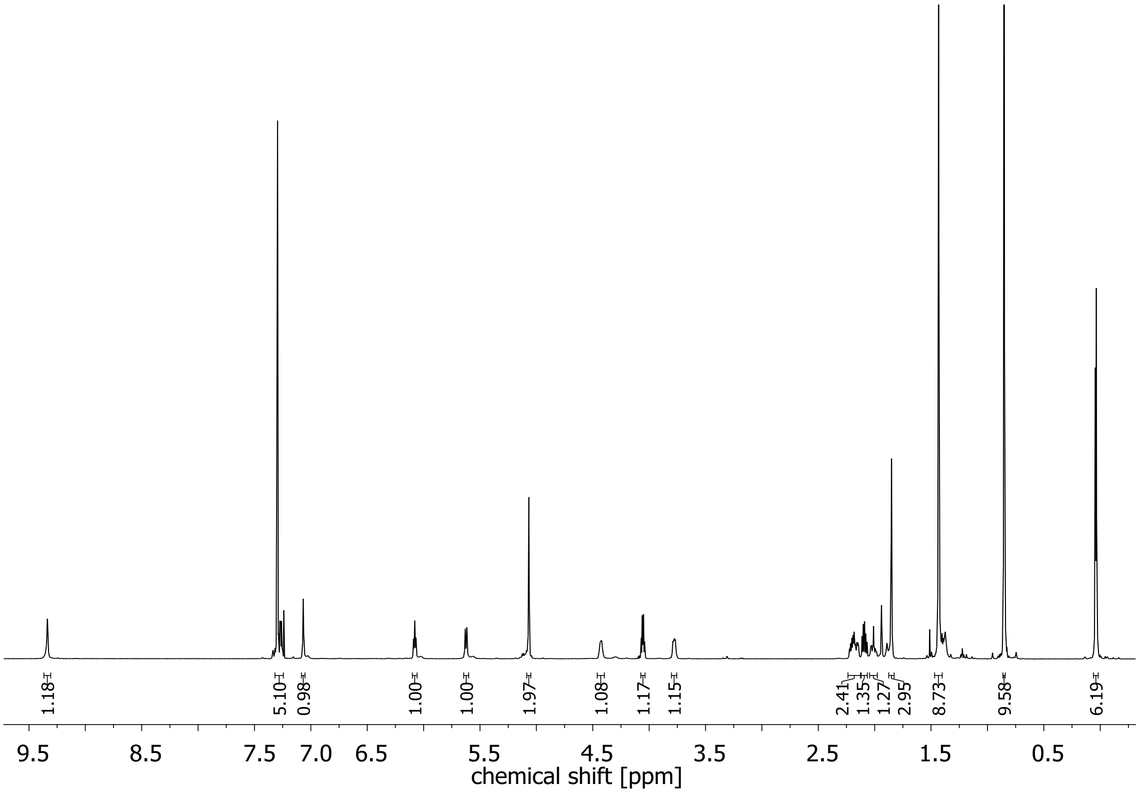

^{1}H NMR spectrum of (*R*)-**20** (300 MHz, $CDCl_3$)

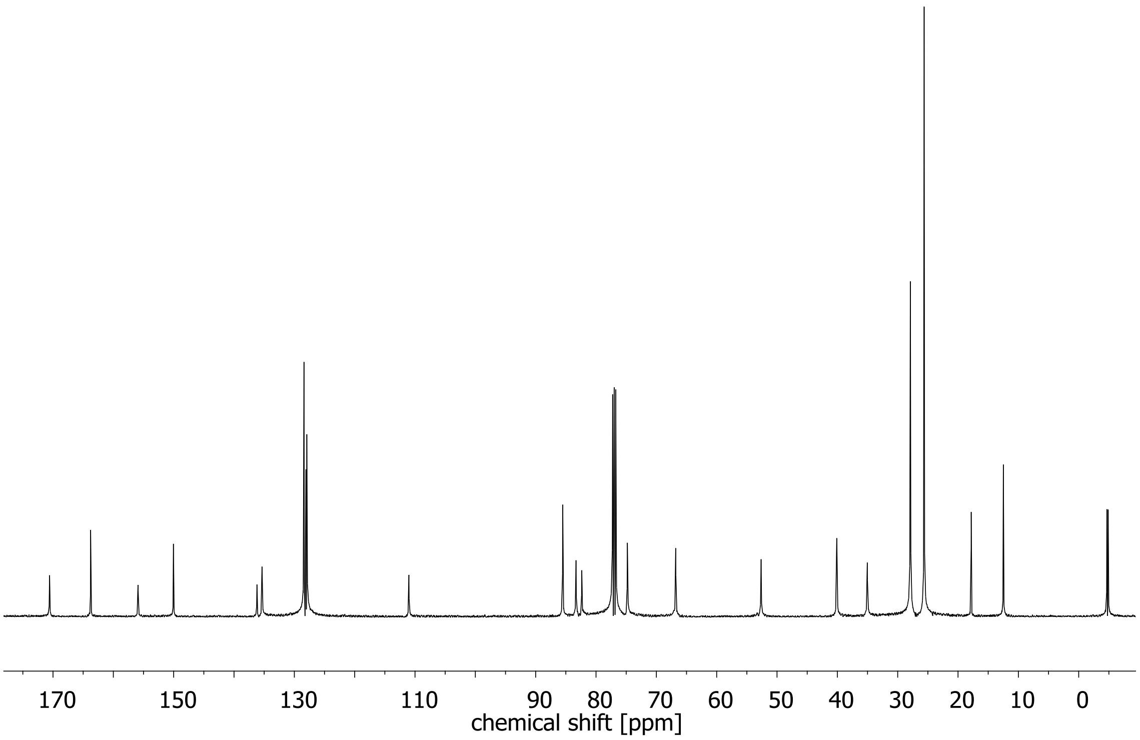

^{13}C NMR spectrum of (*R*)-**20** (75 MHz, $CDCl_3$)

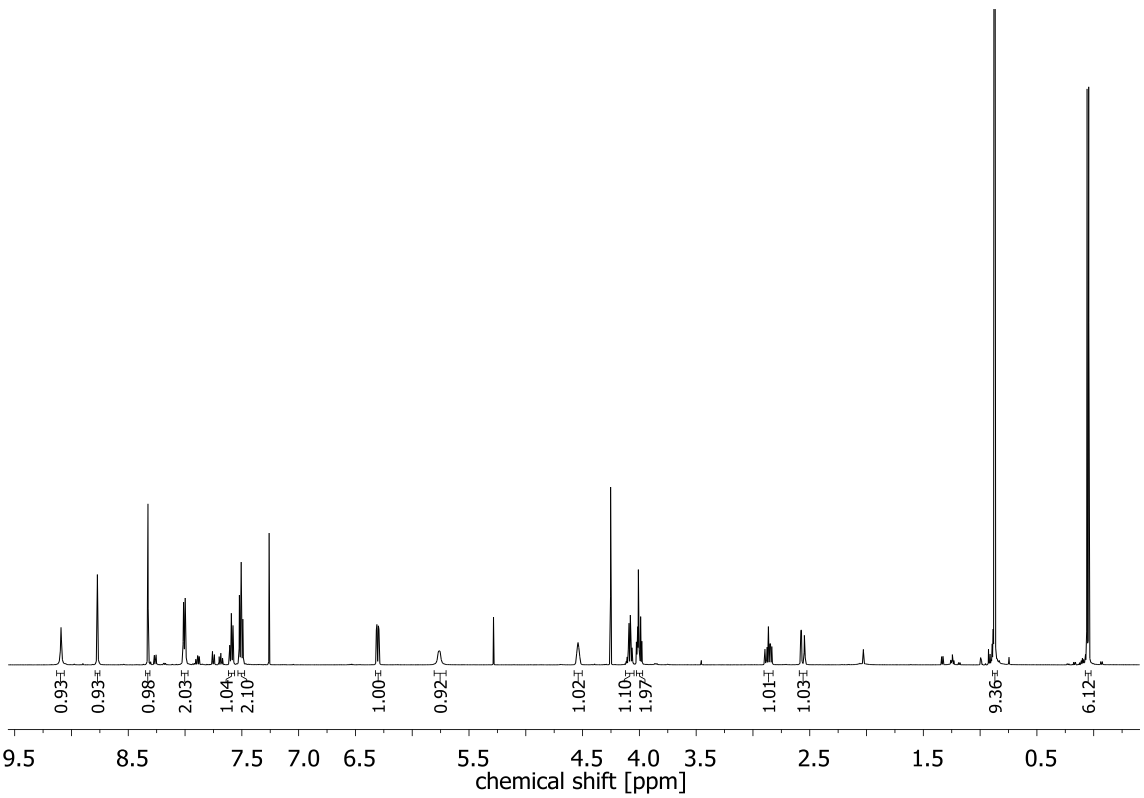

^{1}H NMR spectrum of **24** (300 MHz, $CDCl_3$)

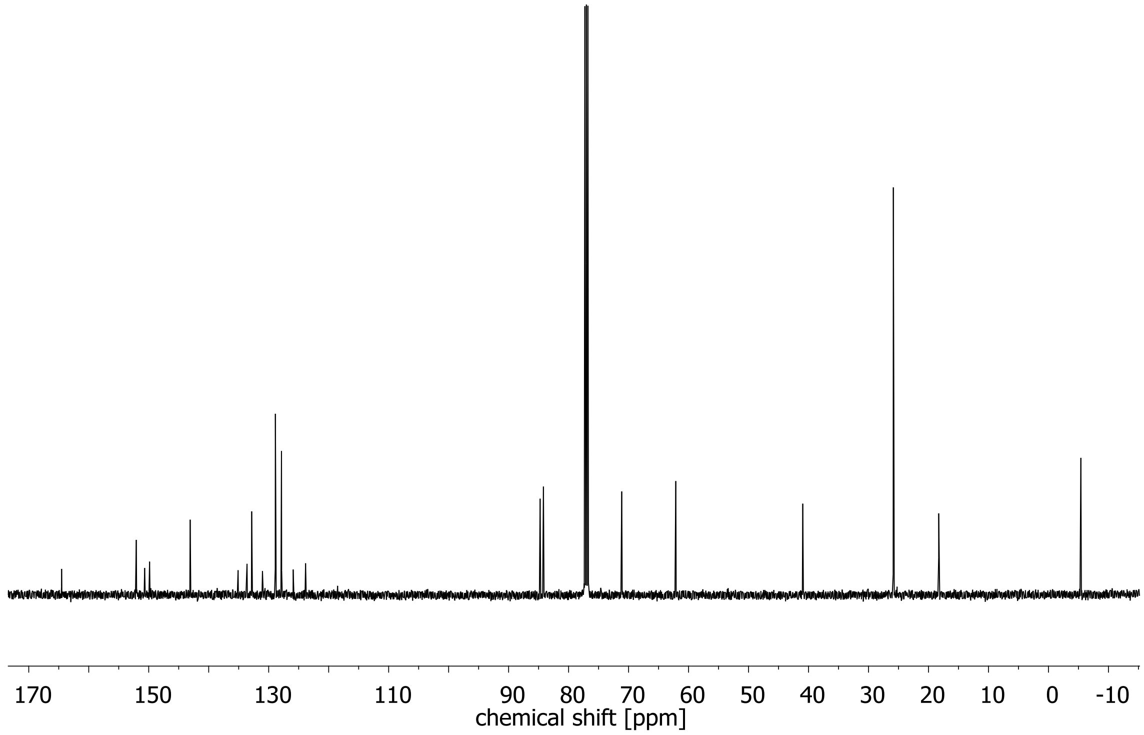

^{13}C NMR spectrum of **24** (75 MHz, $CDCl_3$)

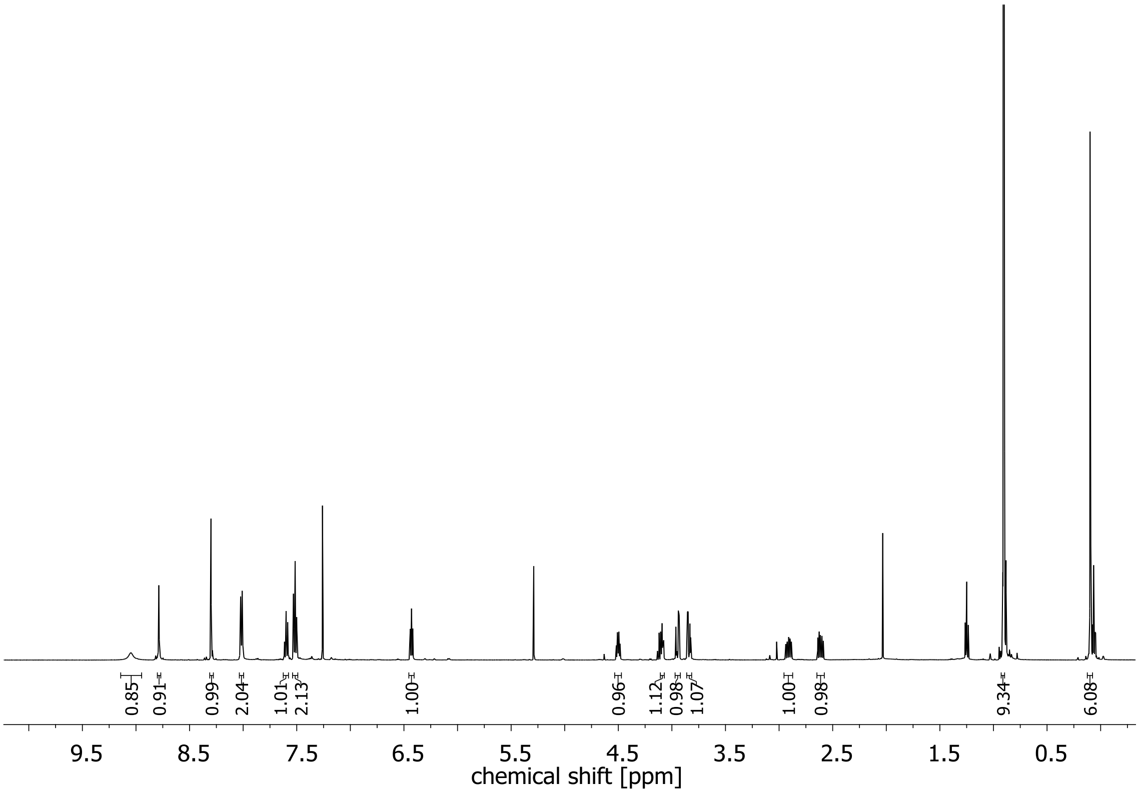

^{1}H NMR spectrum of **25** (300 MHz, $CDCl_3$)

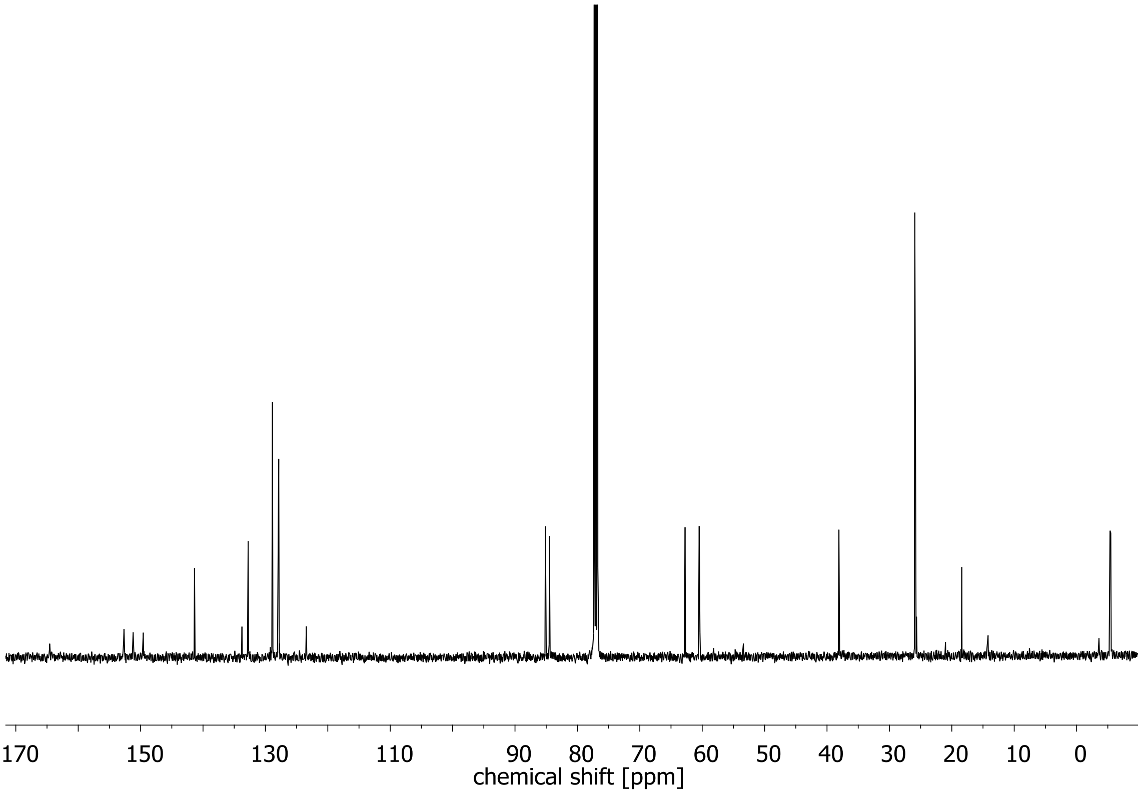

^{13}C NMR spectrum of **25** (75 MHz, $CDCl_3$)

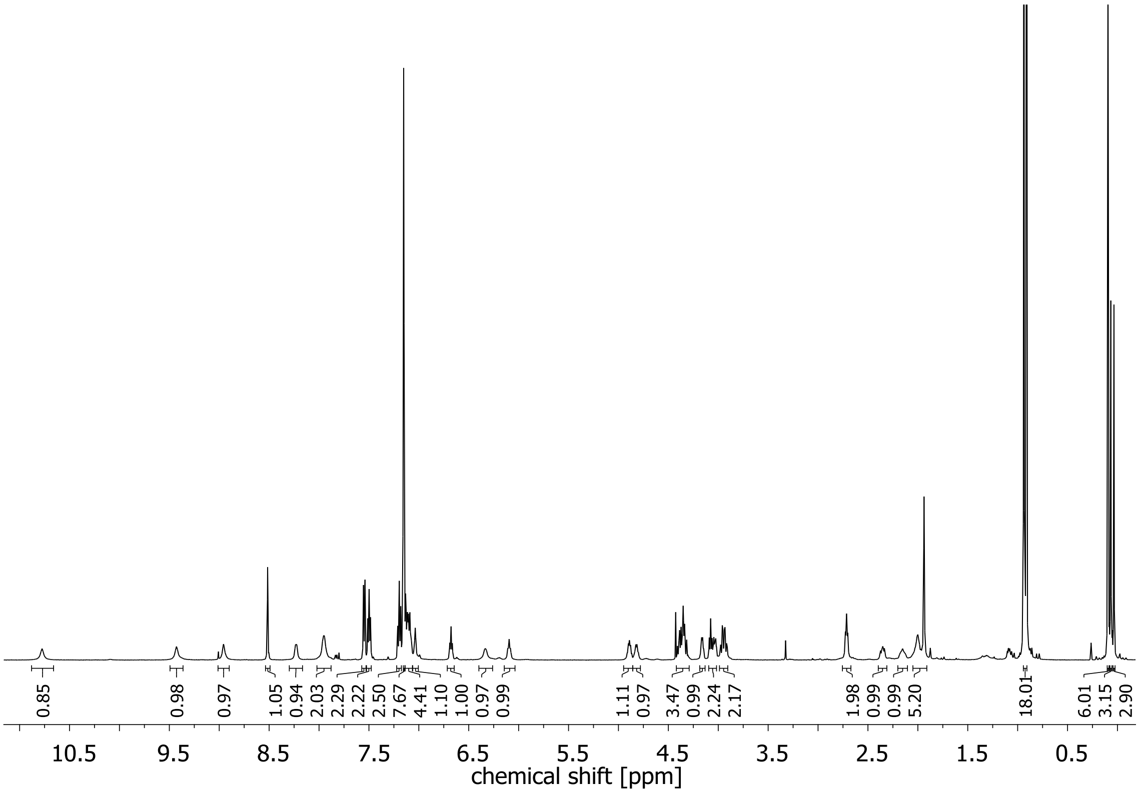

^{1}H NMR spectrum of (*S*)-**26** (500 MHz, C_6D_6, 70 °C)

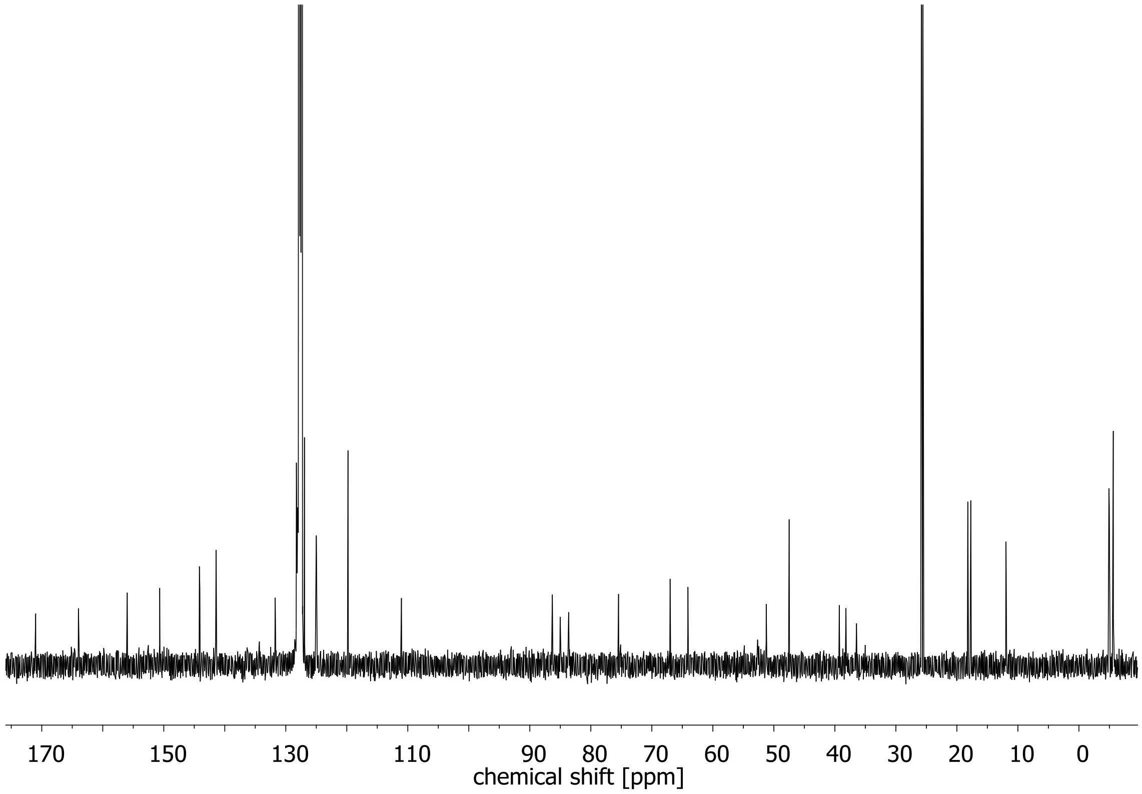

^{13}C NMR spectrum of (*S*)-**26** (126 MHz, C_6D_6, 70 °C)

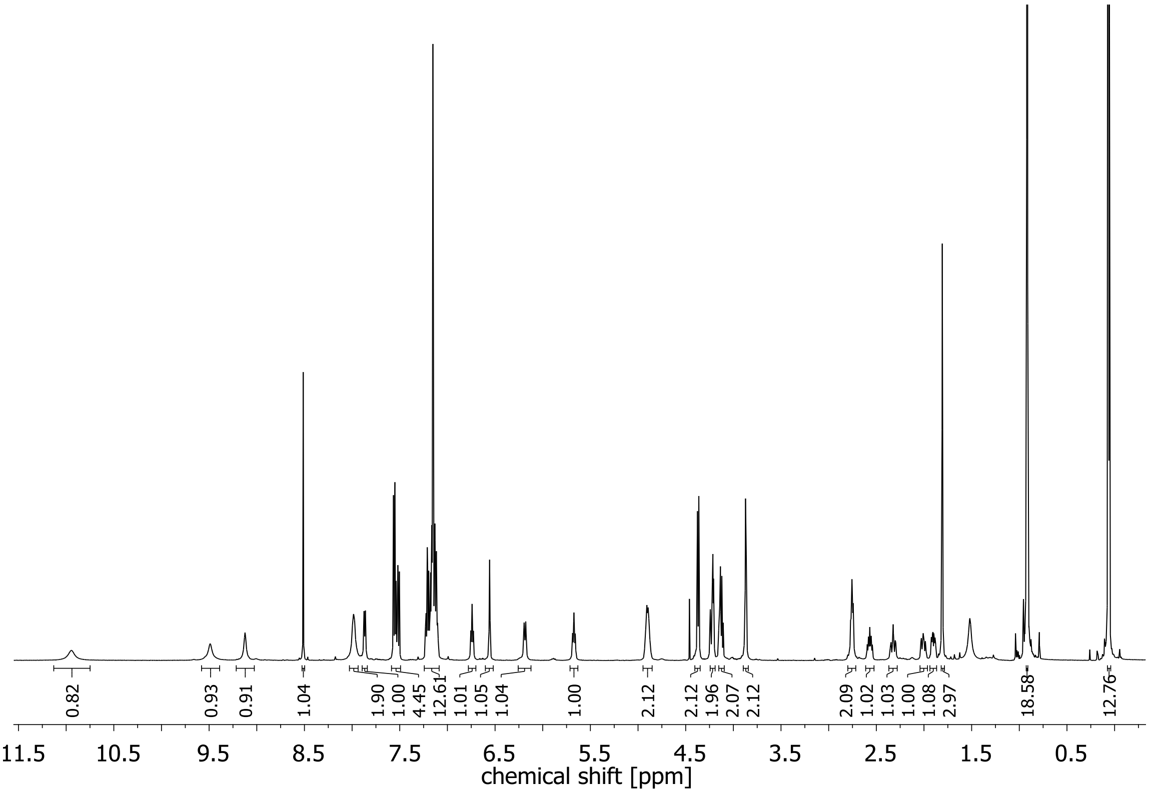

^{1}H NMR spectrum of (*R*)-**26** (500 MHz, C_6D_6, 70 °C)

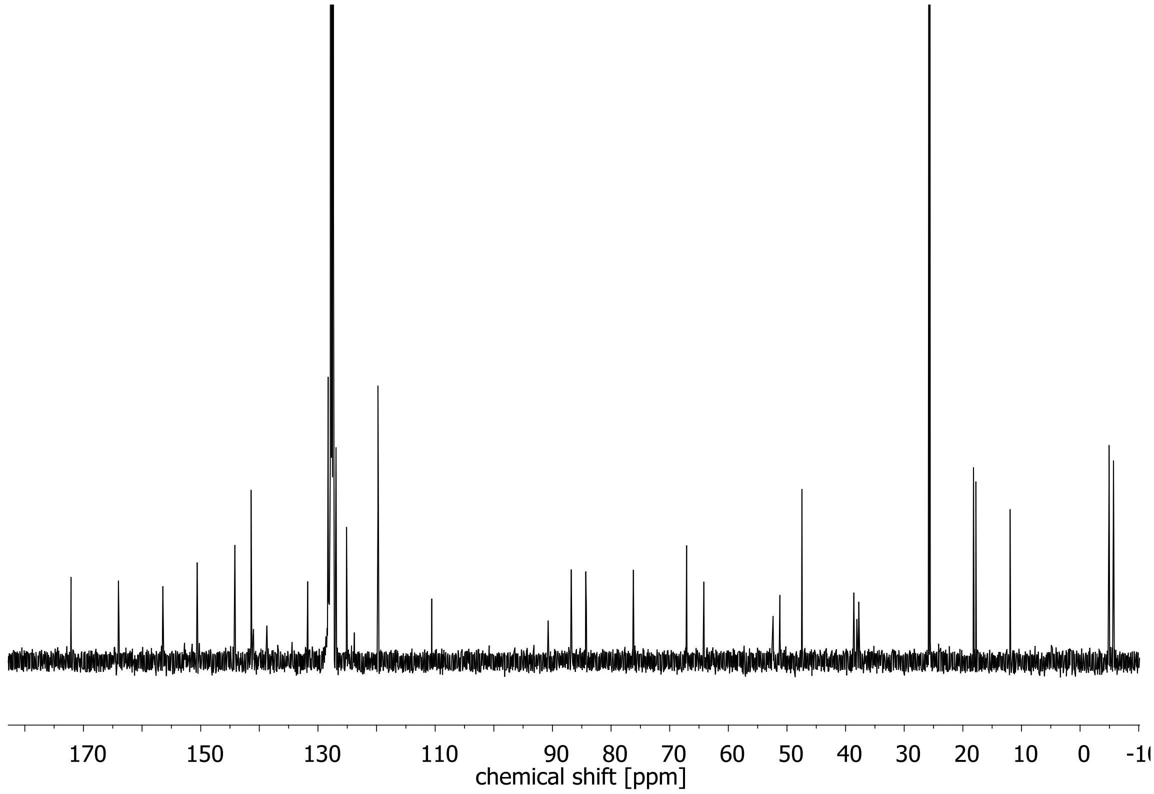

^{13}C NMR spectrum of (*R*)-**26** (126 MHz, C_6D_6, 70 °C)

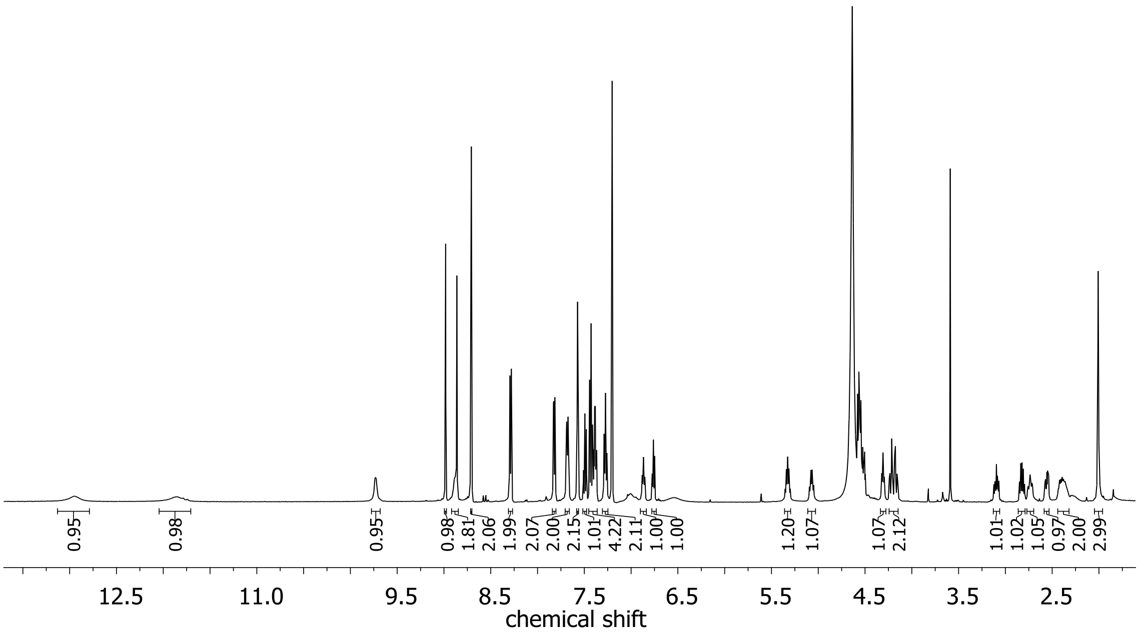

^{1}H NMR spectrum of (*S*)-**27** (500 MHz, pyridine-d$_5$, 50 °C)

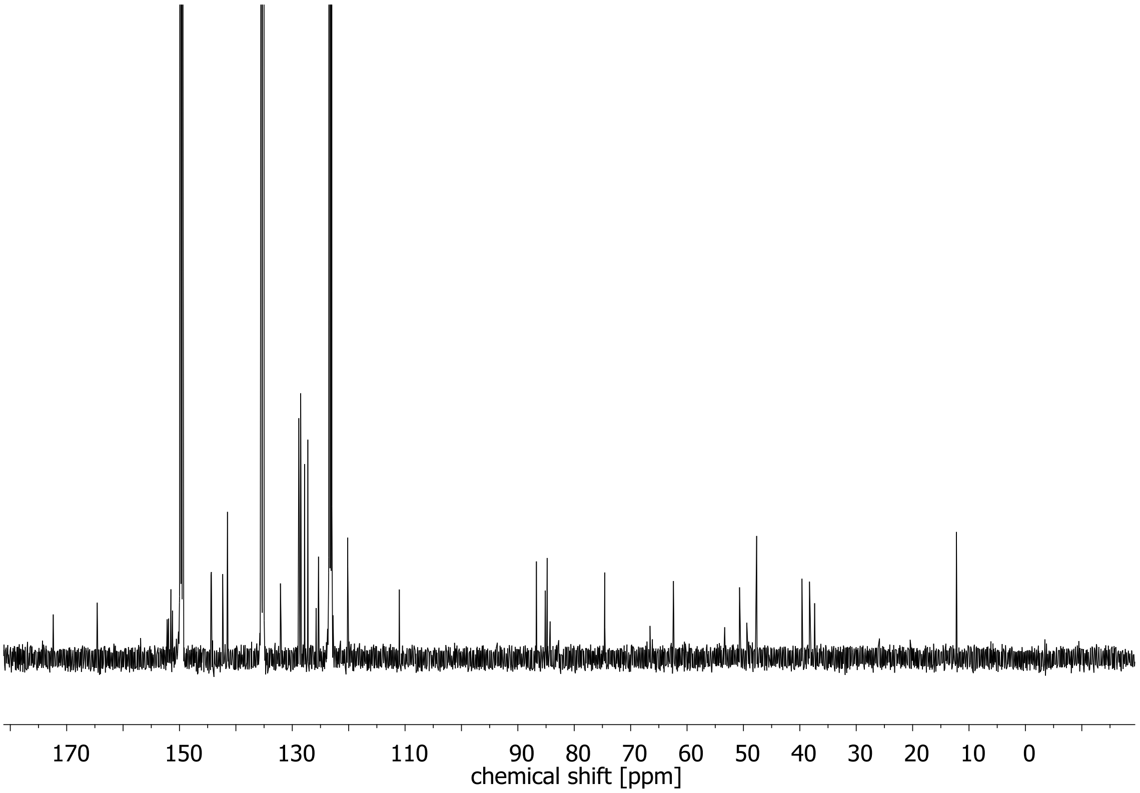

^{13}C NMR spectrum of (*S*)-**27** (126 MHz, pyridine-d$_5$, 50 °C)

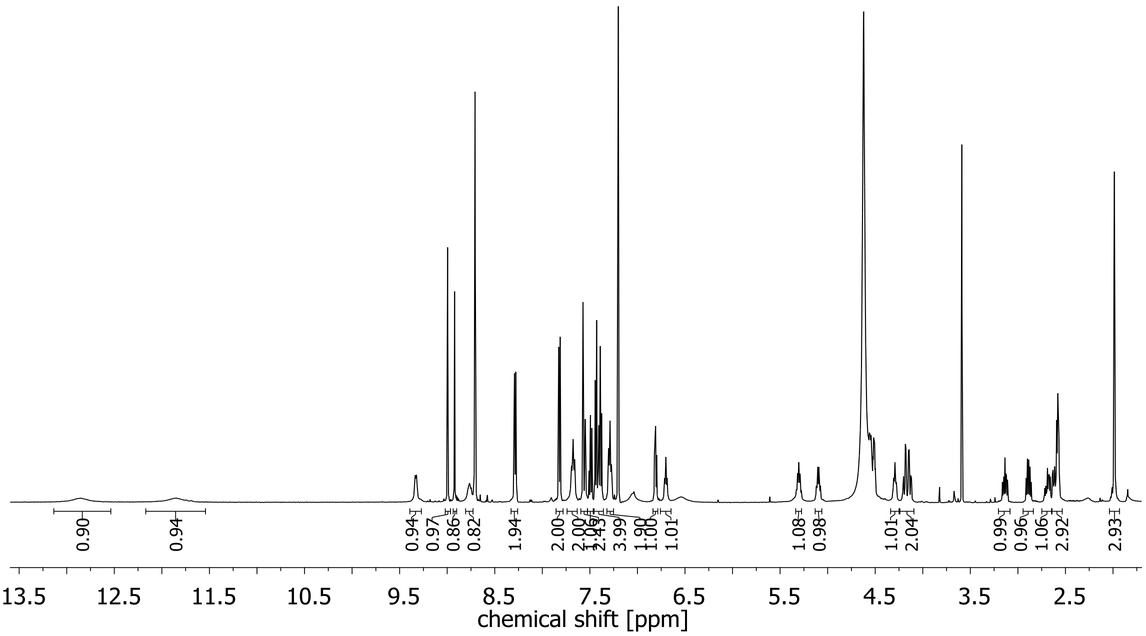

^{1}H NMR spectrum of (*R*)-**27** (500 MHz, pyridine-d_5, 50 °C)

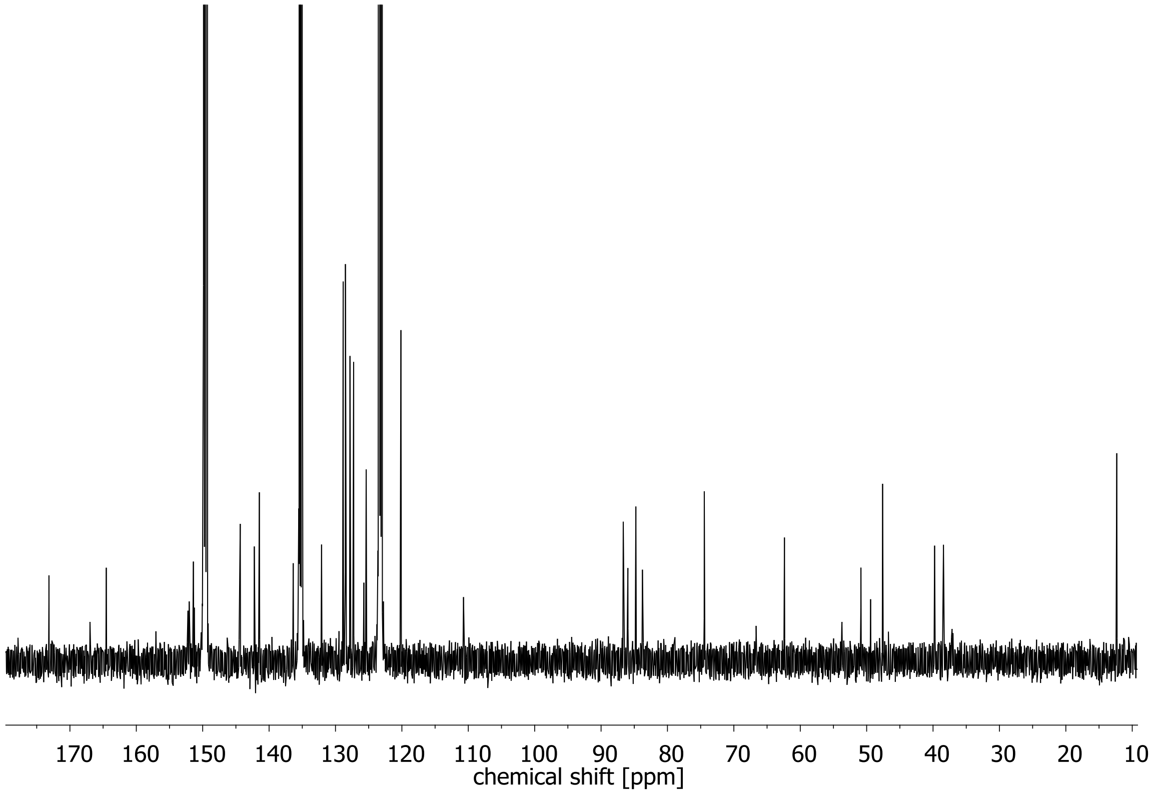

^{13}C NMR spectrum of (*R*)-**27** (126 MHz, pyridine-d_5, 50 °C)

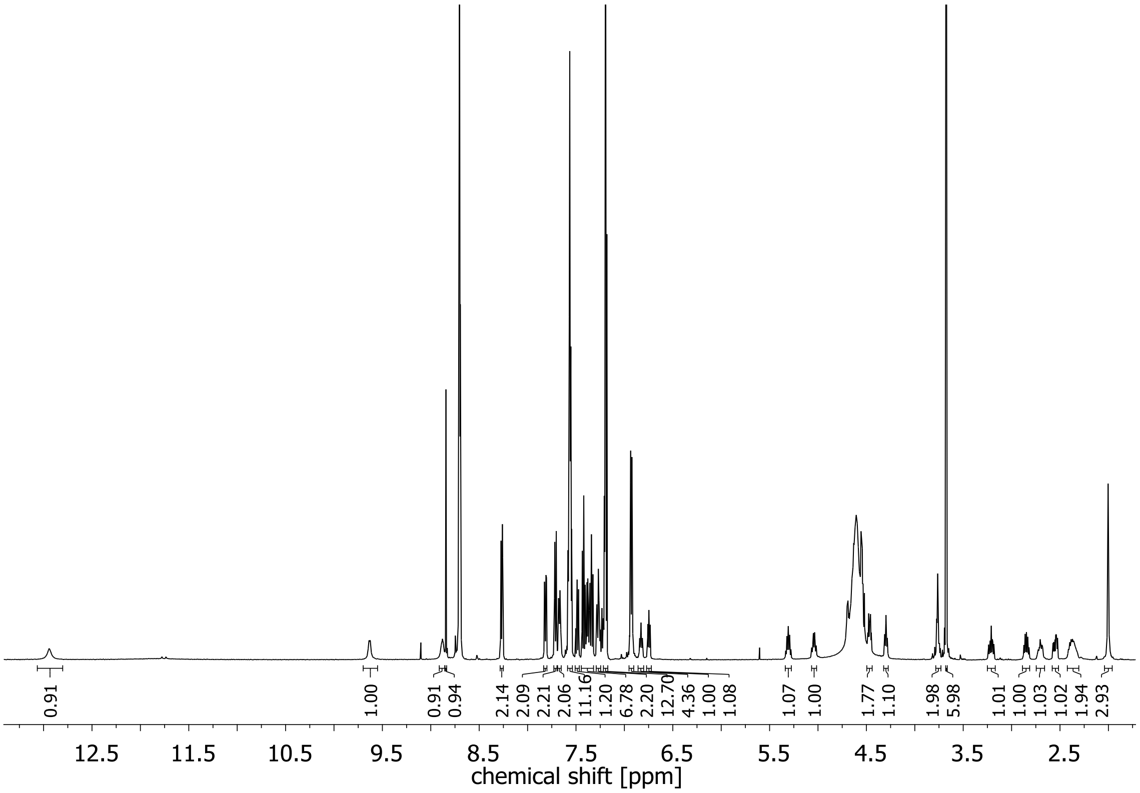

^{1}H NMR spectrum of (*S*)-**28** (500 MHz, pyridine-d_5, 50 °C)

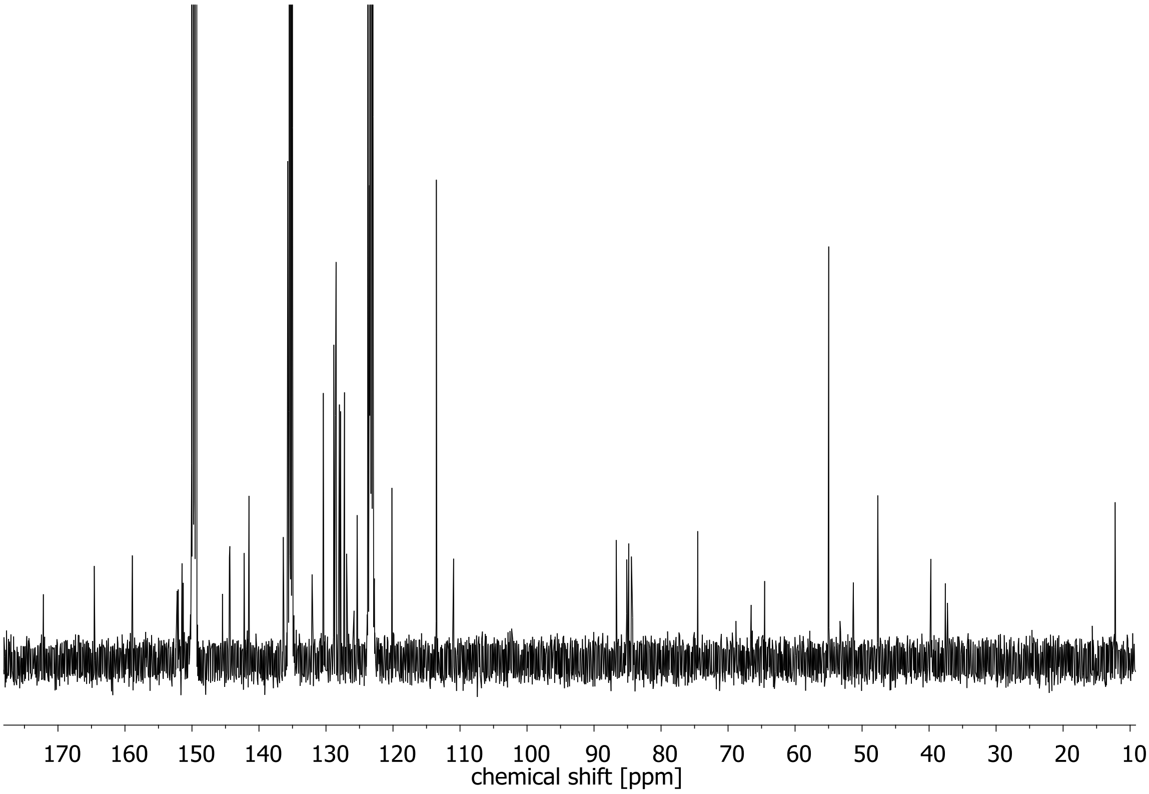

^{13}C NMR spectrum of (*S*)-**28** (126 MHz, pyridine-d_5, 50 °C)

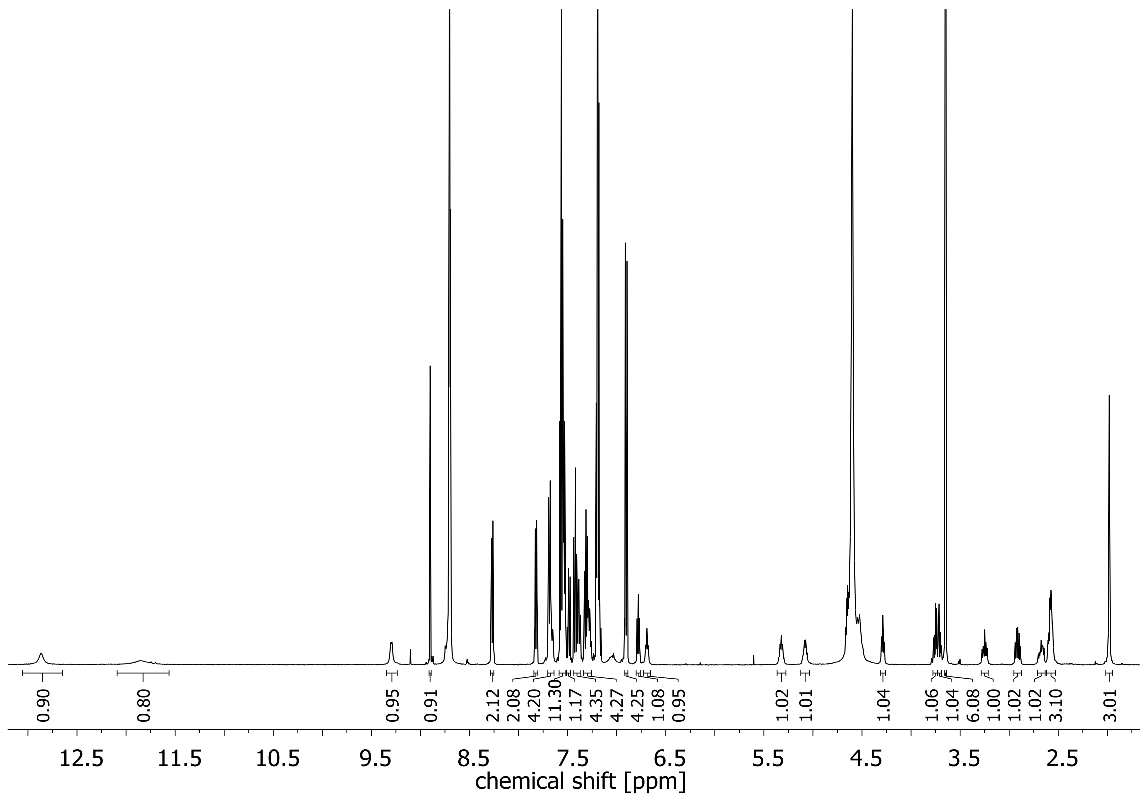

^{1}H NMR spectrum of (*R*)-**28** (500 MHz, pyridine-d_5, 50 °C)

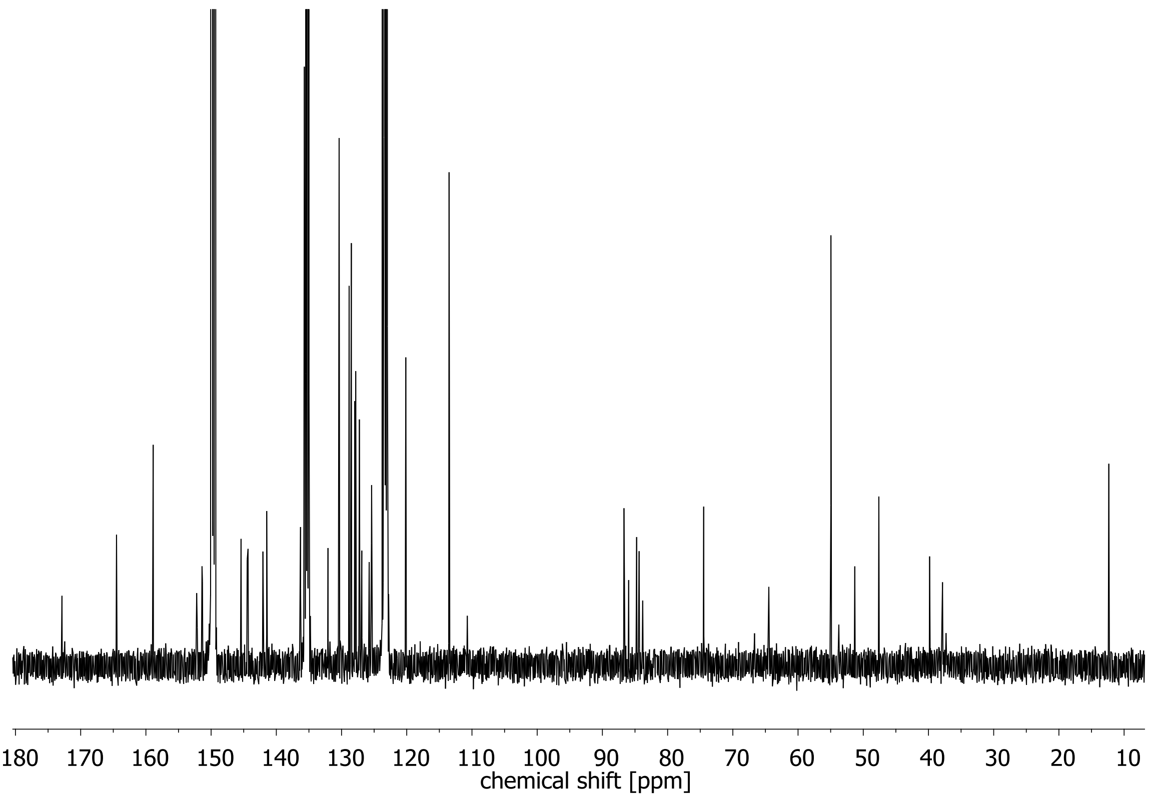

^{13}C NMR spectrum of (*R*)-**28** (126 MHz, pyridine-d_5, 50 °C)

Oligonucleotide synthesis and analytical data of oligonucleotides

Automated synthesis of oligonucleotides

The syntheses of oligonucleotides were performed on a H-8 synthesizer (K & A). For each oligonucleotide synthesis, columns filled with nucleoside-charged CPG resin (200 nmol 5'-*O*-DMTr-nucleoside/g matrix, 3prime) were used. Anhydrous MeCN and CH_2Cl_2 were used as solvents. For the cleavage of DMTr protecting groups, the resin was purged with 3% trichloroacetic acid in anhydrous CH_2Cl_2. The removal of the acid was carried out by purging with anhydrous MeCN. The activation of the phosphoramidite functionality was effected by a 0.25 M benzylthiotetrazole solution in anhydrous MeCN. The coupling time for standard phosphoramidites was 2 min and for NAA-modified building blocks (*S*)-**7** and (*R*)-**7** 4 min. Oxidation of P(III)-species was attained by alkaline iodine solution (20 mM I_2 in pyridine/water 9:1). For the capping of residual 5'-OH-groups, a mixture of solution A (10% Ac_2O, 10% pyridine, 80% THF) and solution B (16% 1-methylimidazole in THF) was used. After completion of the synthesis, the oligonucleotides were cleaved from the solid support with concomitant removal of the Fmoc and β-cyanoethyl protecting groups by reacting the oligonucleotide-charged solid support with 25% aq. NH_3/EtOH (3:1) at 55 °C for 20 h. The thus obtained suspension was filtered and the filtrate was concentrated in vacuo. The resultant residue was dissolved in 750 μL water. For purification of this crude oligonucleotide solution, a volume containing ~ 40 nmol crude oligonucleotide was applied to gel electrophoresis (0.7 mm, 20% polyacrylamide). The oligonucleotide-containing segments of the gel were visualized by UV-light (260 nm) and separated from the rest of the gel. Oligonucleotides were extracted from the gel by incubating each gel segment in 300 μL TEN-buffer (1.0 M TRIS, 0.5 M EDTA, 3.0 M NaCl) at 0 °C for 16 h. The thus obtained TEN-solutions were diluted with 900 μL EtOH and stored for 20 min at -80 °C for precipitation.

Centrifugation at 4 °C and careful removal of the supernatant gave the precipitated pure oligonucleotides.

Analytical data of oligonucleotides

UV spectra of oligonucleotide solutions were measured on a Varian (Cary 100 Bio) within a range Δλ of 320–190 nm. The concentration of the oligonucleotide solutions was ~ 2.0 µM. ESI mass spectra of oligonucleotides were measured in the negative mode on a Thermo Fisher LTQ XL. For the measurements, aqueous 25 µM oligonucleotide solutions with 30% MeCN and 5% NEt_3 were used. Analytical HPLC: the purity analysis of modified and unmodified oligonucleotides by HPLC was performed on a GE Äktapurifier composed of a Dionex P580 HPLC pump, a Dionex ASI-100 fraction sampler, a heating device for the column and a Dionex UV170U UV-detector with four UV–vis channels. For the seperation of oligonucleotides, a Dionex DNAPac PA100 anion exchange column (4 × 250 mm) was used with a flow rate of 1 mL/min and a temperature of 80 °C or 60 °C. The oligonucleotides were eluted using a gradient of 0–60% (during 45 min, for **30**) or 0–40% (during 45 min, for **31**) of eluent B (25 mM TRIS-HCl, 0.5 M $NaClO_4$, 6 M urea, pH 8) in eluent A (25 mM TRIS-HCl, 6 M urea, pH 8). Oligonucleotides were detected by the absorption at λ = 260 nm, and retention times t_R [min] are not corrected.

Table S1. Retention times of synthesized oligonucleotides (HPLC).

no.	sequence	NAA-6'-config.	retention time [min]
1	5'-GGCACGG **AxT AxT** TTTTGGCACGG-3' **30**	*S*	33.3*
2	5'-GGCACGG **AxT AxT** TTTTGGCACGG-3' **31**	*R*	38.7**

* Gradient of eluent B 0-60% during 45 min.

** Gradient of eluent B 0-40% during 45 min.

Table S2. Mass spectral data of synthesized oligonucleotides.

no.	sequence	NAA-6'-config.	calculated	found
1	5'-GGCACGG **AxT AxT** TTTTGGCACGG-3' **30'**	*S*	6760.6	6758.2
2	5'-GGCACGG **AxT AxT** TTTTGGCACGG-3' **31**	*R*	6760.6	6758.6

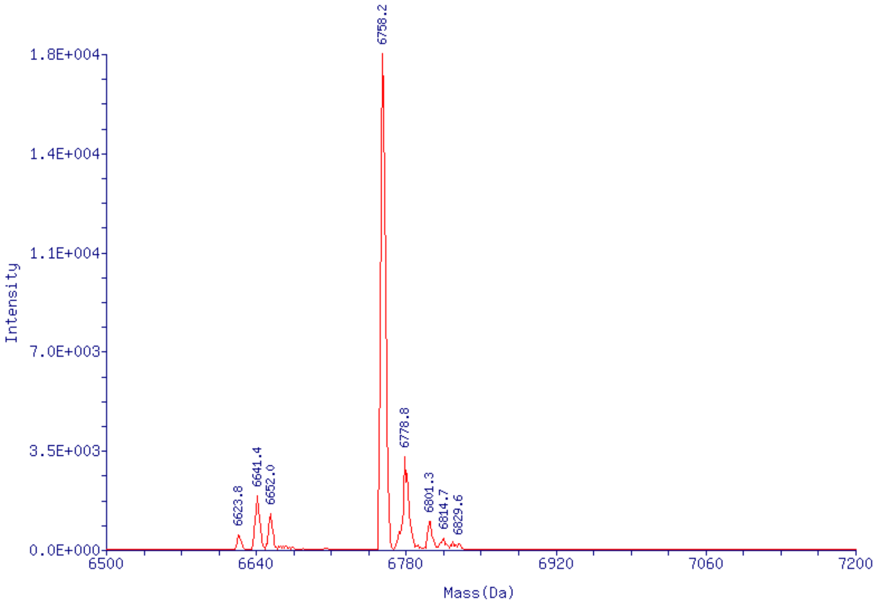

Mass spectrum of **30** (5'-GGCACGGA**x**TA**x**TTTTTGGCACGG-3', **x** = (6'*S*)-NAA)

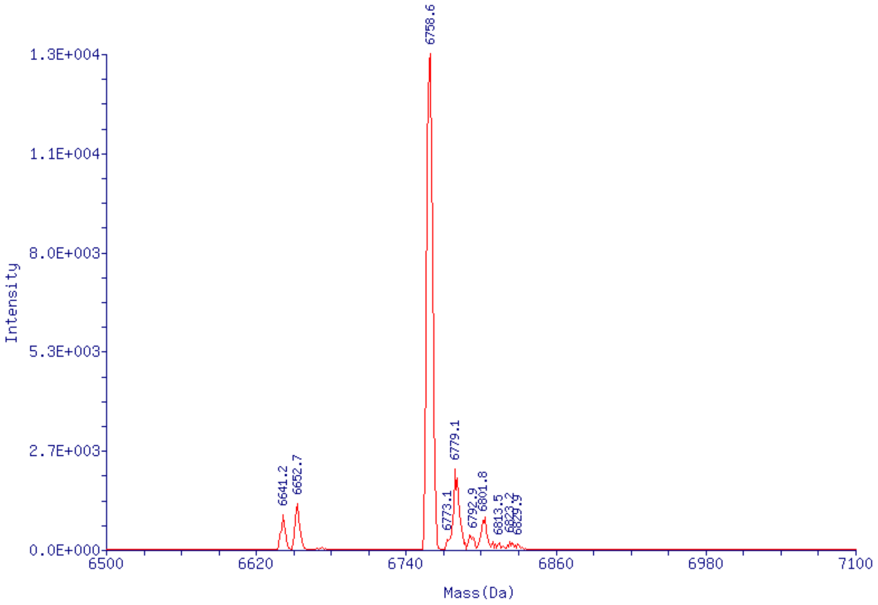

Mass spectrum of **31** (5'-GGCACGGA**x**TA**x**TTTTTGGCACGG-3', **x** = (6'*R*)-NAA)

References

S1 Schmidtgall, B.; Spork, A. P.; Wachowius, F.; Höbartner, C.; Ducho, C. *Chem. Commun.* **2014**, *50*, 13742-13745.

S2 Schmidt, U.; Lieberknecht, A.; Schanbacher, U.; Beuttler, T.; Wild, J. *Angew. Chem.* **1982**, *94*, 797-798; *Angew. Chem. Int. Ed.* **1982**, *21*, 776-777.

S3 Schmidt, U.; Lieberknecht, A.; Wild, J. *Synthesis* **1984**, 53-60.

S4 Schmidt, U.; Wild, J. *Liebigs Ann. Chem.* **1985**, 1882-1894.

S5 Hamzavi, R.; Dolle, F.; Tavitian, B.; Dahl, O.; Nielsen, P. E. *Bioconjugate Chem.* **2003**, *14*, 941-954.

S6 Eisenhuth, R.; Richert, C. *J. Org. Chem.* **2008**, *74*, 26-37.
